# VEGF‐FGF Signaling Activates Quiescent CD63^+^ Liver Stem Cells to Proliferate and Differentiate

**DOI:** 10.1002/advs.202308711

**Published:** 2024-06-17

**Authors:** Fei Chen, Kunshan Zhang, Minjun Wang, Zhiying He, Bing Yu, Xin Wang, Xinghua Pan, Yuping Luo, Shoujia Xu, Joseph T.Y. Lau, Chunsheng Han, Yufang Shi, Yi E. Sun, Siguang Li, Yi‐Ping Hu

**Affiliations:** ^1^ Department of Cell Biology Basic Medical College Second Military Medical University (Naval Medical University) Shanghai 200433 China; ^2^ Stem Cell Translational Research Center School of Medicine and the Collaborative Innovation Center for Brain Science Tongji University Shanghai 200065 China; ^3^ Department of Laboratory Medicine and Pathology University of Minnesota Minneapolis MN 55455 USA; ^4^ Department of Genetics School of Medicine Yale University New Haven CT 06520 USA; ^5^ Shanghai Baixian Biotechnology co., Ltd Shanghai 201318 China; ^6^ Department of Molecular and Cellular Biology Roswell Park Comprehensive Cancer Center Buffalo NY 14263 USA; ^7^ State Key Laboratory of Stem Cell and Reproductive Biology Institute of Zoology Chinese Academy of Sciences Beijing 100101 China; ^8^ Child Health Institute of New Jersey Robert‐Wood Johnson Medical School New Brunswick NJ 08901 USA; ^9^ Department of Psychiatry and Biobehavioral Sciences University of California Los Angeles Los Angeles CA 90095 USA

**Keywords:** lineage tracing, liver injury, liver stem cells, single‐cell RNA‐seq

## Abstract

Understanding the liver stem cells (LSCs) holds great promise for new insights into liver diseases and liver regeneration. However, the heterogenicity and plasticity of liver cells have made it controversial. Here, by employing single‐cell RNA‐sequencing technology, transcriptome features of Krt19^+^ bile duct lineage cells isolated from Krt19CreERT; Rosa26R‐GFP reporter mouse livers are examined. Distinct biliary epithelial cells which include adult LSCs, as well as their downstream hepatocytes and cholangiocytes are identified. Importantly, a novel cell surface LSCs marker, CD63, as well as CD56, which distinguished active and quiescent LSCs are discovered. Cell expansion and bi‐potential differentiation in culture demonstrate the stemness ability of CD63^+^ cells in vitro. Transplantation and lineage tracing of CD63^+^ cells confirm their contribution to liver cell mass in vivo upon injury. Moreover, CD63^+^CD56^+^ cells are proved to be activated LSCs with vigorous proliferation ability. Further studies confirm that CD63^+^CD56^−^ quiescent LSCs express VEGFR2 and FGFR1, and they can be activated to proliferation and differentiation through combination of growth factors: VEGF‐A and bFGF. These findings define an authentic adult liver stem cells compartment, make a further understanding of fate regulation on LSCs, and highlight its contribution to liver during pathophysiologic processes.

## Graphical abstract

Single‐cell RNA sequencing analysis reveals and describes a stem cell population in the biliary epithelial cells of the liver. Liver stem cells contain quiescent (CD63^+^CD56^−^) and active states (CD63^+^CD56^+^). Quiescent liver stem cells express receptors VEGFR2 and FGFR1, which can be further activated through VEGF and FGF signaling, and participate in the liver injury repair.

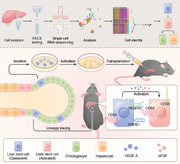

## 1 Introduction

The liver, an organ central in maintaining systemic homeostasis, is endowed with the extraordinary innate ability to self‐renewal.^[^ ^1^, ^2^ ^]^ Canonically, tissue stem cells provide the foundation for tissue homeostatic maintenance and tissue repair after injury by replenishing the organ with new cells to resume function.^[^ ^3^, ^4^ ^]^ However, in the liver mature adult hepatocytes can replenish both hepatocyte and cholangiocyte lineages.^[^ ^1^, ^2^, ^5^, ^6^ ^]^ Therefore, many investigators have thought that adult liver homeostasis and regeneration are achieved solely by self‐replication of terminally differentiated hepatocytes and does not require contributions from presumptive adult liver stem cells (LSCs).^[^ ^7^, ^8^, ^9^, ^10^, ^11^ ^]^ Adults LSCs with the potential to replenish both hepatocyte and cholangiocyte lineages were thought to exist in Canals of Hering (CoH) and peribiliary glands (PBG), but the contribution of LSCs in maintaining tissue homeostasis and/or liver regeneration after injury remains poorly understood.^[^ ^1^, ^12^ ^]^

Under certain types of severe injury such as toxic chemical (e.g., DDC, 3,5‐diethoxycarbonyl‐1,4‐dihydro‐collidine; TAA, Thioacetamide; 2‐AAF, 2‐Acetylaminofluore) induced severe hepatic injury or irreversible block to hepatocytes replication (e.g., p21 over‐expression) induced liver damage in rodents, proliferating duct‐like structures were observed in the portal area of the liver, indicative of a new form of regeneration conducted by biliary epithelial cells.^[^ ^13^, ^14^, ^15^ ^]^ Some of these biliary epithelial cells (BECs) which are Krt19^+^, also exhibit hepatocyte signature.^[^ ^16^, ^17^ ^]^ These hybrid BEC‐hepatocyte cells are believed to be bi‐potential for hepatic and cholangiocytic differentiation,^[^ ^18^ ^]^ a requisite stem cell feature. In separate studies, cells isolated using “stemness” markers such as EpCAM, Sox9, and Lgr5 also appeared to be localized within the biliary tree including CoH. These cells are also Krt19^+^ and possess bipotential‐differentiative ability in vitro.^[^ ^13^, ^19^ ^]^ Taken together, these reports support the notion that stem‐like cells reside within the adult liver and contribute to the maintenance and repair of the liver. Nevertheless, characterization of adult LSCs and their progenies, as well as the signals regulating the cell fate determination remained elusive. This failure is due to the lack of specific molecular markers for the LSCs, as well as the heterogeneity of the microenvironment where the presumptive LSCs reside. Moreover, the different liver injury models used to elicit LSCs activation and repair have generated additional confusion and contentious debates regarding the true identity of LSCs and their function.^[^ ^1^, ^16^, ^18^ ^]^

In this study, GFP‐labeled cells from *Krt19CreERT*; *Rosa26R‐GFP* reporter mouse livers were isolated and analyzed by single‐cell RNA‐sequencing for their individual transcriptional states. Distinct cell groups including BECs and hepatocytes in the liver were identified, including three clusters of BECs. A novel LSC‐specific cell surface marker, CD63, was found and characterized. An additional marker, CD56, which can distinguish between quiescent and active LSCs was also isolated, and signals driving stem cell activation was also identified. The present study lays a new foundation to the understanding of LSCs in liver homeostasis and injury‐repair, which is broadly relevant to studying liver disease as well as tissue stem cells in the other systems.

## 2 Results

### 2.1 Single‐Cell Transcriptomic Analysis Reveals that Krt19^+^ BECs Contain Liver Stem Cells

**Figure 1:**
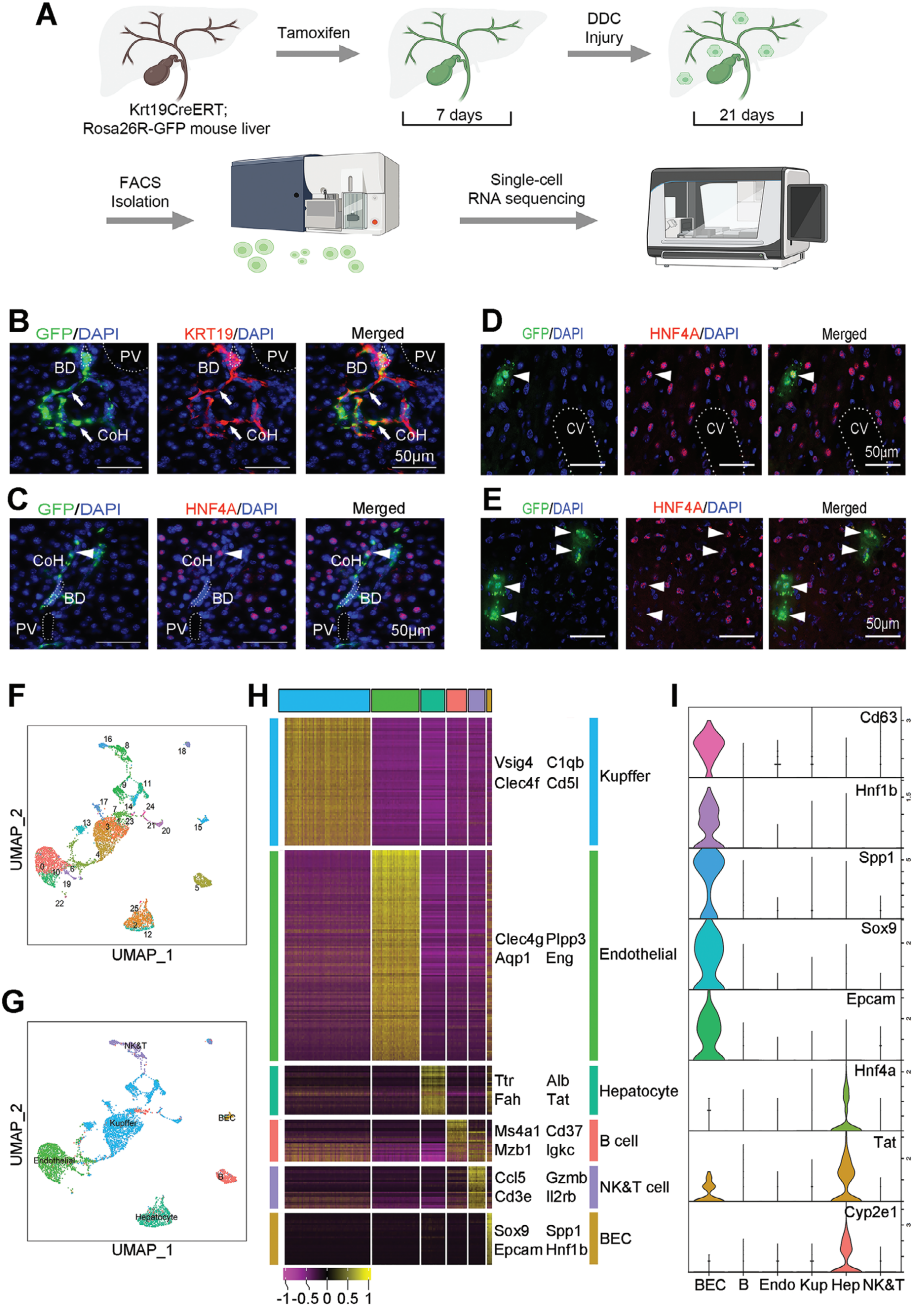
Single‐cell transcriptomes revealed different types of Krt19 lineage cells. A) Schematic diagram for experiments procedure. *Krt19CreERT*; *Rosa26R‐GFP* reporter mice are injected with tamoxifen and given a DDC‐diet for liver injury. GFP^+^ cells in the liver are isolated by FACS and then are performed single‐cell RNA sequencing and analysis. B,C) Representative staining images of livers from *Krt19CreERT*; *Rosa26R‐GFP* mice showed expression of GFP, Krt19 or Hnf4a at day‐7 (B) and day‐14 (C) after TM injection. Nuclei were counterstained DAPI. Arrows point to Canal of Hering (CoH). D,E) Direct fluorescence of GFP combined with staining for Hnf4a in both non‐injury (D) and CCl_4_ injury livers (E). Arrowheads point to hepatocytes. BD, bile duct. PV, portal vein. CoH, Canal of Hering. CV, central vein. Scale bar, 50 µm. F) UMAP plot of Sample clustering revealed 25 cell groups from 7879 single cells. Each group is individually color‐coded. G) Cell type inferred from transcriptomic similarity with six major cell types, including hepatocytes, biliary epithelial cells (BEC), B cells, NK & T cells, Kupffer cells, Endothelial. H) Heatmap of cluster marker genes, color‐coded by cell types (top and right). Columns denote cells; rows denote genes. I) Violin plots display the expression of representative cell‐type markers in six cell types. Y axis is expression levels. Colors represent different genes.

It is postulated that adult LSCs express Krt19 and are located in Canals of Hering (CoH). However, not all Krt19^+^ BECs are LSCs. To identify the adult LSCs from Krt19^+^ cells in CoH, we leveraged the fundamental bipotential ability of true LSCs to differentiate into both hepatocytes and cholangiocytes.^[^ ^1^, ^2^, ^16^ ^]^ *Krt19CreERT* knock‐in mice crossed with *Rosa26R‐GFP* reporter mice, possessing bi‐potential cells in the liver,^[^ ^14^, ^15^, ^17^ ^]^ was used to visualize Krt19 lineage cells (**Figure** **1**A). No GFP^+^ cells were detected without tamoxifen (TM) treatment. 7 days after TM treatment, GFP and Krt19 doubly positive cells were predominantly restricted to the portal triad area (Figure S1A,B, Supporting Information), where portal vein (PV), hepatic artery, biliary duct (BD) are located. Cholangiocytes express Krt19 were highly enriched on BD structures and were negative for Hnf4a, aSMA, and albumin (Figure S1C, Supporting Information). GFP also labeled Canals of Hering (CoH), where putative LSCs resided (Figure 1B, indicated by arrows). By 14 days after TM induction, few GFP^+^ cells expressing the hepatic marker, Hnf4a, were observed (Figure 1C, arrowhead), indicating limited hepatic lineage development of Krt19 expressing BECs in normal physiological conditions. In contrast, many more GFP^+^ hepatic cells were observed 7 days after tetrachloromethane (CCl_4_) induced acute injury (Figure 1D,E). These results indicate that the Krt19‐GFP labeled BECs contain putative LSCs, which are activatable for liver repair upon injury.

To understand the nature and relationship of the Krt19‐lineage cells, GFP^+^ cells were isolated and subjected to single‐cell transcriptome analysis. Earlier studies proposed that under certain types of hepatic stress, such as injury resulting from DDC insult, LSCs could be activated for the liver repair.^[^ ^14^, ^15^, ^16^ ^]^ Therefore, the DDC injury model was used to reveal the full spectrum of LSCs activation and downstream differentiation. 7876 individual cells at 7 days after TM injection followed by 21 days after DDC injury were analyzed by 10x Genomics single‐cell RNA‐seq (scRNA‐seq) technology (Figure 1A). Based on the Uniform Manifold Approximation and Projection (UMAP) analyses of the transcriptomics data, 25 clusters of cells were identified from the GFP^+^ cells (Figure 1F), and they were projected onto 6 types identified from other experiments including hepatocytes, BECs, Kupffer cells, NK and T cells, endothelial, and B cells^[^ ^17^, ^18^ ^]^ by using scmap (Figure 1G).^[^ ^20^ ^]^ The identities of these clusters were revealed by interrogating the expression of known liver cell marker genes (*Alb*, *Tat*, *Fah* for hepatocytes and *Epcam*, *Sox9*, *Spp1* for cholangiocytes) among a large number of genes highly expressed in each of the 6 corresponding major cell types (Figure 1H,I).

**Figure 2:**
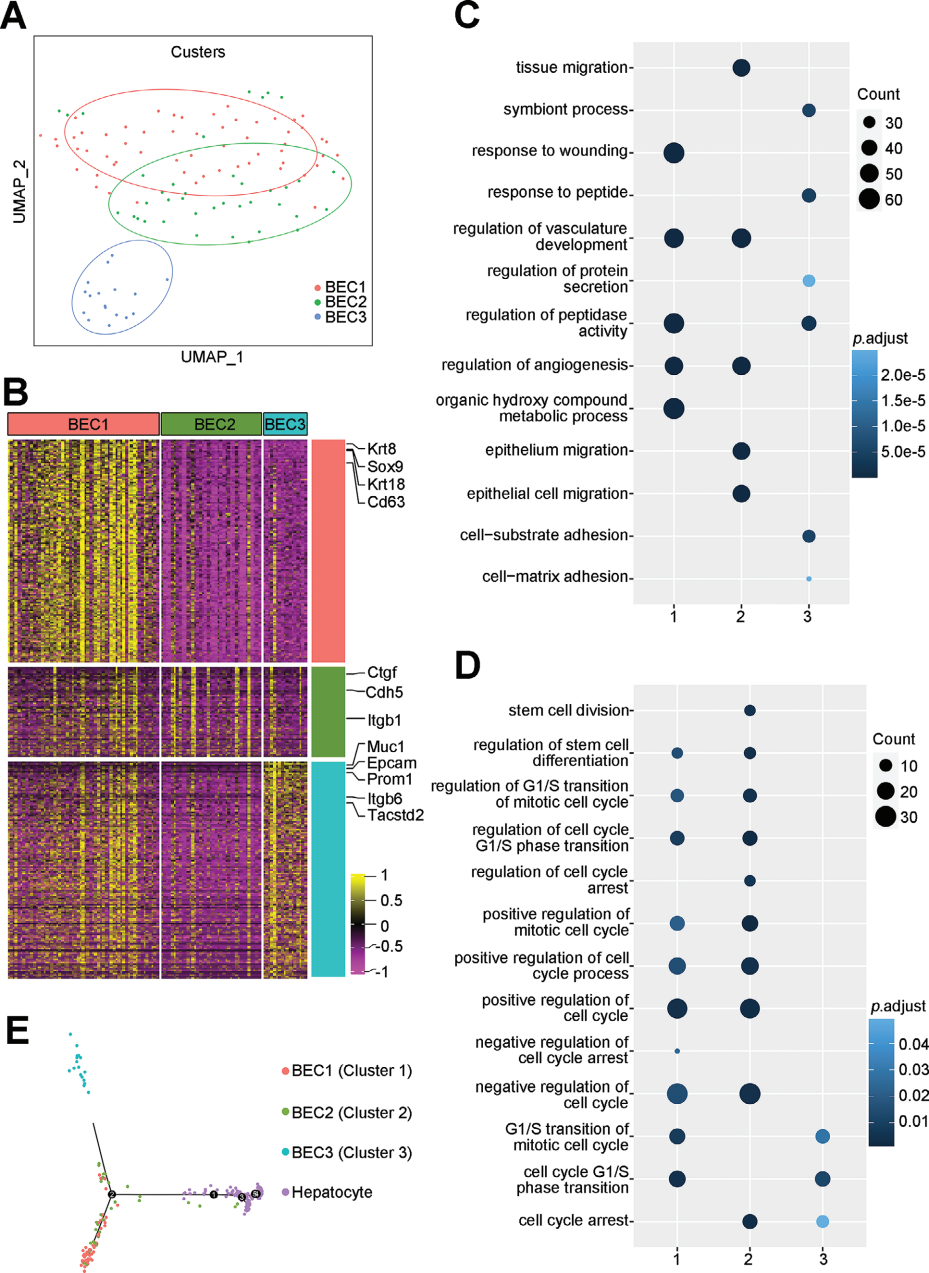
Identification of subgroups in BECs reveals stem‐like cells. A) UMAP plot shows three subgroup in BECs, each subgroup were in different colors. B) Heatmap of the top expressed genes in three BEC subgroups. C) Enriched GO analysis of the up‐regulated genes in three BEC subgroups. Colored circle indicates P values of GO terms in each subgroup. D) Stem cells and cell cycle related GO terms in each subgroup. E) Trajectory analysis of the potentially bi‐direction differentiation model from subgroup 1 into cholangiocytes and hepatocytes.

Previous studies suggest that bi‐potential liver stem cells reside in the cholangiocytes.^[^ ^14^, ^15^, ^16^ ^]^ Therefore, we focused on BECs to distinguish clusters which may contain liver stem cells. Three sub‐clusters of BECs were identified by a shared nearest neighbor (SNN) modularity optimization‐based clustering algorithm (**Figure** **2**A). Genes preferentially expressed in each sub‐cluster were identified. Many of the well‐known cholangiocytic markers were detected, including *Epcam*, *Sox9*, *Prom1*, etc. (Figure 2B). To define cell types of each sub‐cluster based on gene expression, gene ontology (GO) analysis was carried out. GO terms such as “epithelial cell migration” were enriched in genes specifically expressed in sub‐clusters 1 and 2 suggesting that these two sub‐clusters are likely luminal epithelial cells (Figure 2C). GO terms related to protein transport and epithelial cell polarity such as “regulation of protein secretion” and “cell−substrate adhesion” are enriched in the genes specifically expressed in sub‐cluster 3, indicating that these cells may be functional cholangiocytes (Figure 2C). We observed the enrichment of stem cell‐associated terms in Cluster 1 and 2, but not in Cluster 3, suggesting the presence of LSCs in Clusters 1 and Cluster 2 but not in Cluster 3. Compared to Cluster 1, Cluster 2 was more enriched with cell cycle and proliferation‐related transcripts, suggesting a more actively proliferating progenitor cell population in Cluster 2 (Figure 2D). To further verify the lineage relationship of these clusters, we carried out Pseudo‐timeseries analysis on BECs and hepatocytes was performed to assess the lineage relationships between these clusters. A three‐branched relationship was observed (Figure 2E). Occupying the ends of two of the branches were putatively functional cholangiocytes (Cluster 3 of BECs) at one branch, and hepatocytes at the end of the second branch. The third branch was formed by a majority of Cluster 1 cells. Cluster 2 cells were distributed along all three branches and were more distant from hepatocytes than from the putative functional cholangiocytes. The distribution structure suggest that Cluster 1 cells are LSCs, and Cluster 2 cells are the “transit‐amplifying cells (TAC)”, which is consistent with the concept that LSCs is bi‐potential for differentiating into hepatocytes and cholangiocytes.

### 2.2 Identification of a Novel LSC Cell Surface Marker, CD63

**Figure 3:**
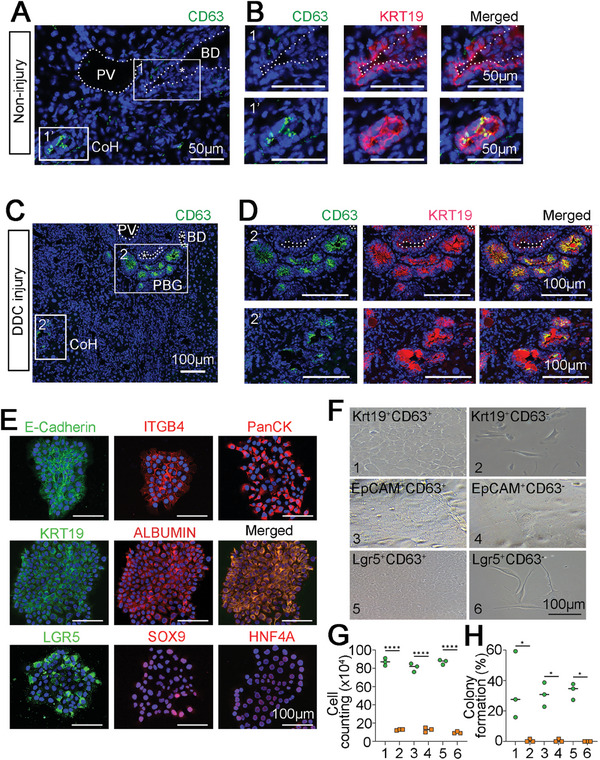
Characterization of CD63^+^ cells in the adult mouse liver. A,B) Representative images showed CD63 and Krt19 expression at the CoH in non‐injury liver. Panel B was magnified view of insert box in panel A co‐staining with Krt19. Scale bar, 50 µm. C,D) Representative images showed CD63 expression in DDC Injury liver. Panel D was CD63 and Krt19 staining of the boxed region in Panel c. Asterisks in panel C indicated bile ducts negative for CD63 but positive for Krt19. Scale bar, 100 µm. E–H) Characterization of CD63^+^ cells in vitro. (E) Representative images showed immunostaining of CD63^+^ single cell‐derived clones by previously reported LSC markers: E‐Cadherin, Krt19, Lgr5 (green) and Itb4, PanCK, Alb, Sox9, Hnf4a (red). Scale bar, 100 µm. Nuclei were counterstained DAPI. (F) Representative phase contrast images showed the growth of Krt19^+^CD63^+^ 1), Krt19^+^CD63^−^ 2), EpCAM^+^CD63^+^ 3), EpCAM^+^CD63^−^ 4), Lgr5^+^CD63^+^ 5), Lrg5^+^CD63^−(^ ^6^ ^)^ cells respectively. Scale bar, 100 µm. G) Quantification of cell numbers in each group. H) Colony formation efficacy of each group. Data are the mean ± SEM. *n* = 3. ^***^ *p* < 0.001. ^*^ *p* < 0.05.

To investigate novel surface markers of liver stem cells, which could be conveniently applied for further study, cluster of differentiation (CD) genes that are highly expressed in Cluster 1 BECs were examined to identify candidates. *Cd63*, a member of tetraspanin family, was the most specifically expressed CD gene in cluster 1 (Figure 2B). We examined whether CD63 could serve as a novel pan LSC marker. In adult liver, Canals of Hering (CoH) and peribiliary gland (PBG) are thought to provide the specialized niche harboring LSCs.^[^ ^21^ ^]^ Immunocytochemistry analyses using antibodies against CD63 were carried out in non‐injury normal mice, in mice with liver damage by a chronic diet containing DDC. In adult non‐injury mice, CD63 was expressed in CoH around the portal triads area (**Figure** **3**A,B). The large bile duct lined by mature cholangiocytes was CD63 negative but Krt19 positive (asterisks in Figure 3A,C), suggesting that CD63 labeled more specifically than Krt19. In DDC injury mice, PBG deep in the duct walls (staining with aSMA, Figure S2A, Supporting Information) was strongly CD63^+^ and Krt19^+^ (Figure 3C,D, top box and panels). No mature hepatocyte or stellate cells expressed CD63 (Figure 3A–D; Figure S2A, Supporting Information). CD63^+^ Krt19^+^ double positive cell abundance was 0.51% ± 0.07%in normal liver, and this population increased to 3.53% ± 0.51% in DDC injured liver. The CD63^+^ cells also expressed other markers such as EpCAM and Lgr5 (Figure S2B,C, Supporting Information).^[^ ^16^, ^19^ ^]^ However, both EpCAM and Lgr5 have broader staining pattern than CD63, indicating CD63 labeling is more specific. In the acute CCl_4_ injury model, the CD63^+^ cells could also be detected in the Krt19^+^ duct area (Figure S2D, Supporting Information). Taken together, these results indicate that CD63 identifies a specific population of cells within CoH and PBG, consistent with the notion that CD63 might be a more specific marker for LSC.

To assess the self‐renewal and differentiation ability of CD63^+^ cells, we used a series of in vitro clonal cultures of cells isolated from total non‐parenchymal cells (NPCs) by FACS. CD11, CD45, and CD68 were used to exclude leukocytes, Kupffer cells, and endothelial cells.^[^ ^16^, ^17^ ^]^ Approximately 0.2% and 0.7% of NPCs from non‐injury and DDC injury livers, respectively, were CD63^+^. These CD63^+^ but CD11/CD45/CD68‐tripple negative cells, hereafter shall be referred to as CD63^+^ cells, were cultured with ScmA medium.^[^ ^22^ ^]^ In culture, CD63^+^ cells from non‐injury or DDC injury mice could form epithelial clones (Figure 3E). The CD63^+^ cell, with a mean diameter of 10 µm, polarized with high nucleus to cytoplasm ratio, and could be expanded >20 passages in culture, displaying a typical S‐shaped growth curve, demonstrating extensive self‐renewal capacity (Figure S3A, Supporting Information). Cultured CD63^+^ cells also express many previously postulated putative markers for LSCs (Figure 3E; Figure S3B, Supporting Information). To confirm whether CD63^+^ cells were superior than other biliary cells/liver stem cells, CD63^+^/CD63^−^Krt19^+^, CD63^+^/CD63^−^EpCAM^+^ and CD63^+^/CD63^−^Lgr5^+^ cells were isolated by FACS. The six populations were plated at identical density to assess their growth ability. CD63^+^Krt19^+^, CD63^+^EpCAM^+^, and CD63^+^Lgr5^+^ cells could form epithelial clones while the other three populations deteriorated (Figure 3F). These results were statistically confirmed by cell counting assay and colony formation experiments (Figure 3G,H). Lgr5 was previously regarded as a liver stem cell marker, therefore Lgr5^+^ liver stem cells were compared with CD63^+^ cells by microarray data. Transcriptome analysis indicated that reported liver stem cells (CD133^+^, Lgr5^+^ and Foxl1^+^ cells) and CD63^+^ cells were clustered together, but were all distinct from mature hepatocytes. CD63^+^ cells and CD63^+^Lgr5^+^ cells show a more similar RNA expression pattern (Figure S3C, Supporting Information).

**Figure 4:**
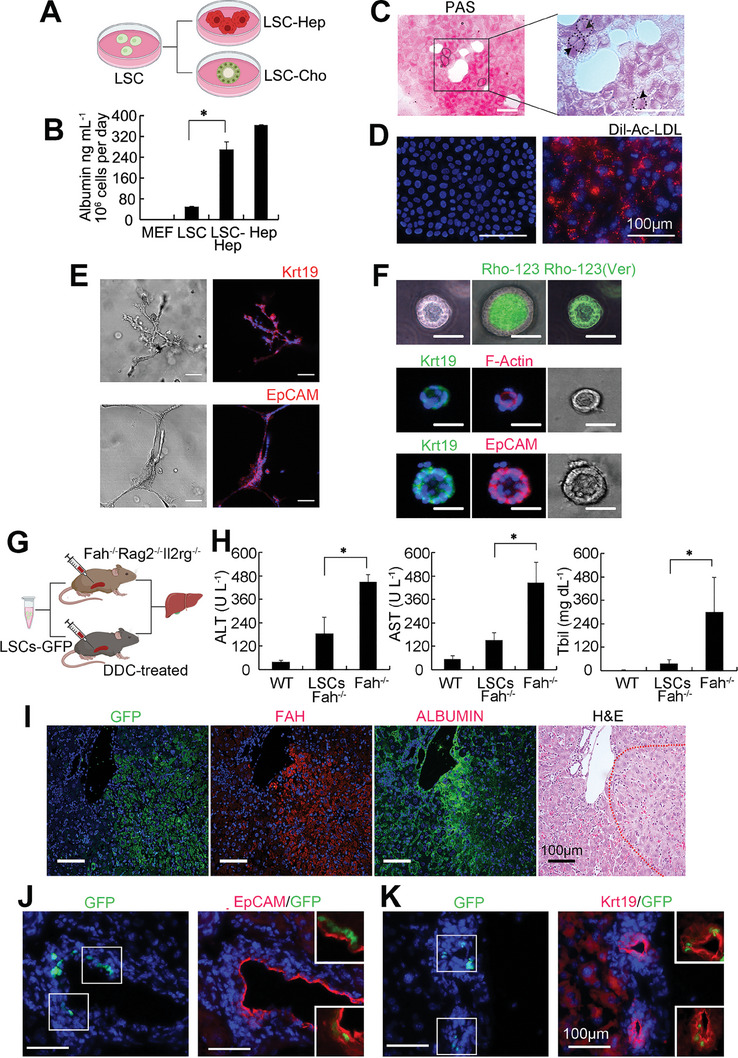
Bi‐potential differentiation of CD63^+^ cells in vitro and in vivo. A–F) Representative data of in vitro differentiation of CD63^+^ cells. A) Schematic procedures of in vitro differentiation. LSCs were cultured in hepatic‐ and cholangiocytic‐ medium, respectively. B) ELISA showed the levels of Albumin secretion and the data were represented as mean ± SD. *n* = 3. ^*^ *p* < 0.05. MEF, mouse embryonic fibroblasts. LSC‐Hep, hepatic induced differentiated cells from LSC. C) PAS staining displayed mature hepatocyte character. Dashed line and arrowhead indicate binucleation cells. Box inserted was high magnified view. Scale bar, 50 µm. D) LSC‐Hep cells could take up fluorescent‐labeled substrate of Dil‐ac‐low‐density lipoprotein (Dil‐Ac‐LDL). Scale bar, 100 µm. E,F) CD63^+^ cells were cultured in cholangiocytic‐differentiation medium. E) Typical branching structures were observed and stained with Krt19 or Epcam (red). Nuclei are counterstained with Dapi (blue). Scale bar, 100 µm. F) Cells were cultured in 3D condition. Confocal microscopy images (top three panels) showed transport of rhodamine 123 (Rho‐123) into the central lumen of a cyst and verapamil (Ver) blocking Rho‐123 transport. The middle and bottom panels show the morphology and staining of the cysts by Krt19 (green), F‐actin (red), Epcam (red). Nuclei are counterstained with Dapi (blue). Scale bar, 50µm. G–K) In vivo assay of CD63^+^ cells after transplantation cells into injured mouse model. G) Schematic procedures of in vivo transplantation. LSCs were labelled with GFP and injected into the spleen of Fah^−/−^Rag2^−/−^Il2rg^−/−^ mouse and DDC‐treated mice, respectively. Livers were harvested and analyzed. H) Serum levels of ALT, AST, and Tbil in each group. WT, wild type mouse. LSCs Fah^−/−^, Fah^−/−^Rag2^−/−^Il2rg^−/−^ mouse transplanted with LSCs. Fah^−/−^, Fah^−/−^Rag2^−/−^Il2rg^−/−^ mouse. Data were represented as mean ± SD. *n* = 3. ^*^ *p* < 0.05. I) Immunofluorescence staining of Fah^+^ area. Panels are serial sections of the same area staining with GFP (green), FAH (red), Albumin (green), H&E. Red dashed line cycled the fah^+^ area. Scale bar, 100 µm. J,K) Transplantation of GPF^+^ cells using DDC injury model. Representative views of GPF^+^ bile duct. Direct fluorescence of GFP combined with staining of EpCAM (J) Krt19 (K). Inserted boxes show the high magnified view of the double positive area. Nuclei was counterstained with Dapi (blue). Scale bar, 100 µm.

To determine the differentiation potential of CD63^+^ putative LSCs, we used a series of classic hepatic and cholangiocytic lineage differentiation assays to demonstrate that CD63^+^ cells are bi‐potential (**Figure** **4**; Figure S4, Supporting Information). For in vitro study, in the hepatic differentiation medium (Figure 4A), CD63^+^ cells could get the functional characteristics of hepatocytes, which include Albumin secretion (Figure 4B), glycogen storage indicated by Periodic Acid‐Schiff (PAS) staining (Figure 4C) and low density lipoprotein uptake (Figure 4D). When these CD63^+^ cells were cultured in the 3D condition with cholangiocytic medium, they formed bile duct‐like structures with branches (Figure 4E). Also, they could organize into a cyst, which could transport of rhodamine 123 (Rho‐123) into the central lumen and block by verapamil (Ver, Figure 4F). CD63^+^Lgr5^+^ cells were also exanimated by these approaches and were proven to be bi‐potential (Figure S4A–E, Supporting Information). Notably, hepatic cells derived from CD63^+^ or CD63^+^Lgr5^+^ cells exhibited elevated ability of Albumin secretion, CYP450 metabolism (Figure S4F–H, Supporting Information). To test whether CD63^+^ cells can repopulated in vivo, we carried out cell transplantation experiments using fumarylacetoacetate hydrolase‐deficient (*Fah* ^−^ *^/^* ^−^) mice lacking mature T, B and NK cells (*Rag2* ^−^ *^/^* ^−^ *Il2rg* ^−^ *^/^* ^−^)^[^ ^19^ ^]^ and DDC injury mice (Figure 4G). CD63^+^ cells were first labelled with GFP. Fah^−/−^Rag2^−/−^Il2rg^−/−^ mice livers were analyzed 12 weeks after cell transplantation. Results indicated that CD63^+^ cells repopulated the damaged liver and differentiated into hepatocytes, which expressed albumin and fumarylacetoacetate hydrolase (Figure 4I). Also, the function of liver was restored indicated by the serum levels of Alanine aminotransferase (ALT), Aspartate aminotransferase (AST) and Total bilirubin (Tbil) (Figure 4H). In the DDC‐diet mice group, the bile duct epithelial cells were damaged. We found that GFP^+^ cells could participate in regeneration of bile duct cells (Figure 4J,K). These confirmed that CD63^+^ cells have the ability to generate functional hepatocytes and cholangiocytes in vivo, in host livers.

**Figure 5:**
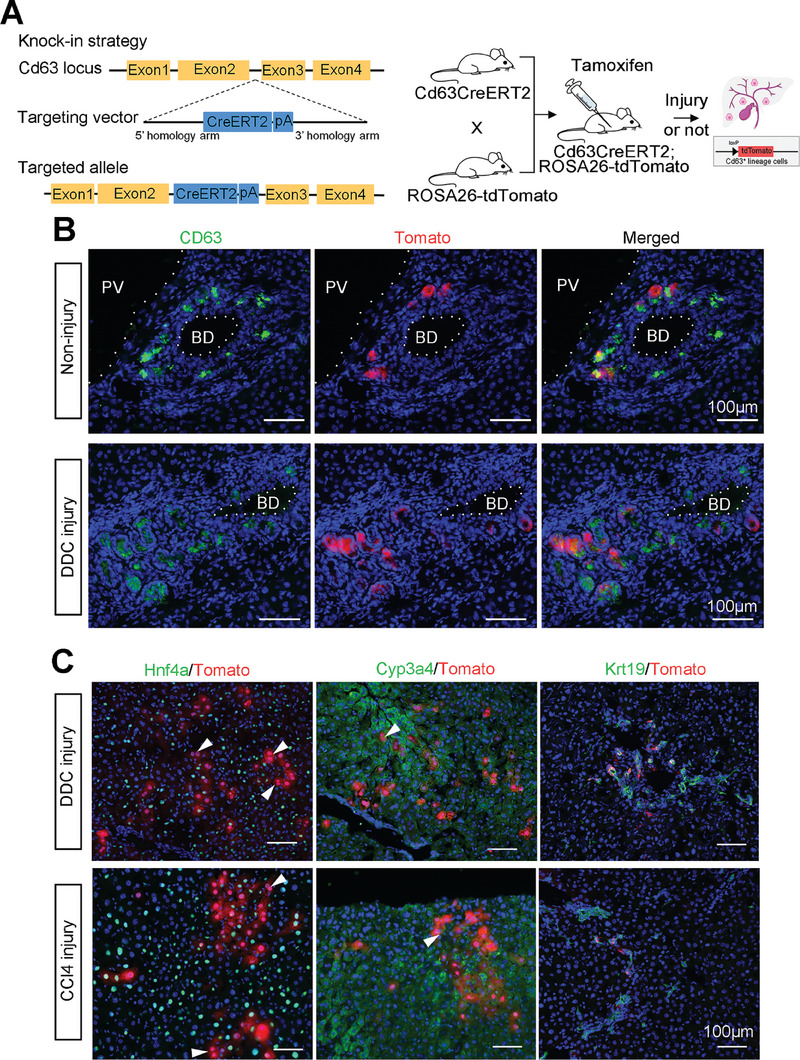
Lineage tracing of CD63^+^ cells in vivo using reporter mice. A) Schematic diagram showed generation of *CD63CreERT2*; *Rosa26‐tdTomato* mouse. The CreERT2‐pA sequence was integrated into the second exon of the Cd63 gene to get *CD63CreERT2* knock‐in mice and then crossed with *Rosa26‐tdTomato* mouse. After TM administration, the CD63^+^ cell and its lineage were labeled with Tomato. B) Representative immunostaining images showed expression of CD63 (green) and Tomato (red) in both non‐injury and DDC injury *CD63CreERT2*; *Rosa26‐tdTomato* mouse liver. C) Representative immunostaining images showed Tomato‐labeled CD63 lineage cells could differentiate into hepatocytes and cholangiocytes, in DDC and CCl_4_ injury liver. Co‐immunostaining of Tomato with Hnf4a or Cyp3a4 indicated hepatocytes (left and middle panels), and Tomato with Krt19 indicated cholangiocytes (right panels). Arrowheads indicated binucleation hepatocytes. Scale bar, 100 µm. Nuclei were counterstained DAPI.

To trace the in vivo lineage development of endogenous CD63^+^ cells, we generated *CD63‐promoter CreERT2* knock‐in mice and crossed with the *Rosa26‐TdTomato* reporter mice. 8‐week‐old *CD63CreERT2*; *Rosa26‐TdTomato* reporter mice were injected with tamoxifen to activate Cre (**Figure** **5**A), and mouse livers were analyzed (Figure 5; Figure S5, Supporting Information). No recombination was detected in *CD63CreERT2*; *Rosa26‐TdTomato* mice without TM (Figure S5A, Supporting Information). The labeling efficiency of the Tomato^+^/CD63^+^ cells in the liver were 31.40% ± 2.79% and 31.29% ± 11.93% in the non‐injury and DDC injury mouse, respectively (Figure 5B). There were no mislabeled hepatocytes at the dose of TM administration (Figure S5B, Supporting Information). In two different liver regeneration models: the DDC injury and CCl_4_ injury, tomato^+^ cells contributed to both hepatocytes and cholangiocytes (Figure 5C). Tomato^+^ hepatocyte contributed to 5.3% ± 0.64% and 5.7% ± 0.83% of the liver in DDC and CCl_4_ models, respectively. The contribution raised to 8.0% ± 0.97% in the chronic CCl_4_ injury model. Both tomato^+^ cells and hepatocytes were isolated for chromosome analysis (Figure S5C, Supporting Information). Results indicated that tomato^+^ cells had normal chromosomal numbers (n = 40).

These data unequivocally demonstrated that CD63^+^ cells were authentic adult LSCs.

### 2.3 Transcriptome Dynamics of Quiescent and Activated Liver Stem Cells

**Figure 6:**
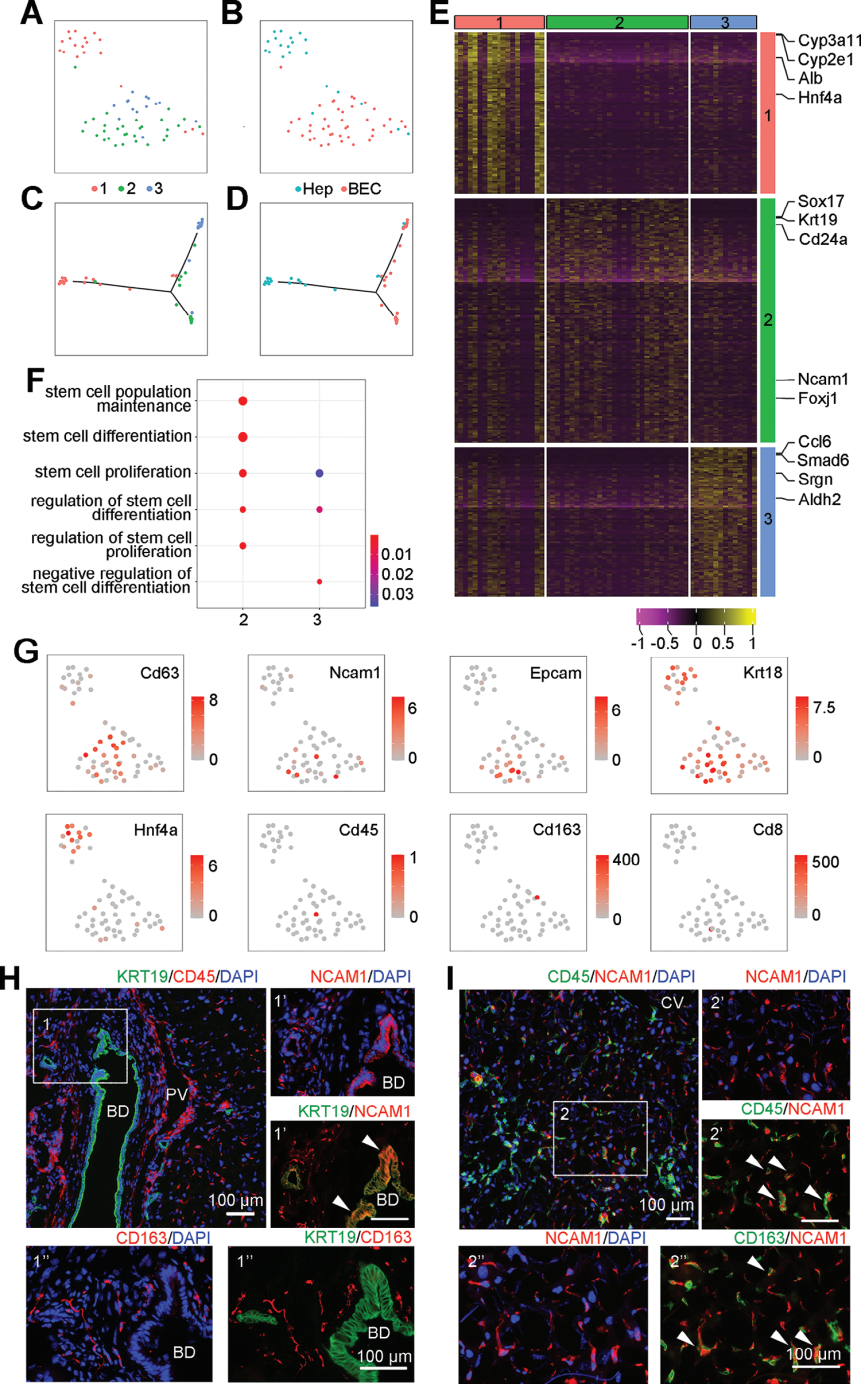
Subgroup analysis of CD63 lineage cells uncovered quiescent and activated liver stem cells. A) TSNE plot of three groups in Cd63 lineage cells. B) Cell type annotated by 10x transcriptomic data. Notice that the group 2 (blue dots) and 3 (green dots) are mainly annotated as BECs. C,D) Trajectory analysis show the potentially development path of Cd63 lineage. The hepatocytes (subgroup 1) were mainly enriched in left branch, while the BECs were divided in two branches. In which, the subgroup 3 dominantly clustered in up‐right, while the subgroup 2 were scattered on right branch. E) Heatmap shows top expressed genes in 3 groups in Cd63 lineage, including hepatocytic markers (Cyp2e1, Alb), cholangiocytic markers (Krt19, Hnf4a). F) Dot plot shows stem cell related GO terms enriched in signature genes of subgroup 2 and 3. No stem cells were enriched in group 1. G) TSNE plots shows expression of different markers. H) Representative immunostaining images show expression of KRT19 (green) and NCAM1, CD45 or CD163 (red) in the liver. I) Immunostaining images showed expression of CD45 or CD163 (green) and NCAM1 (red) in the liver. Inserted boxes and numbers are serial section of staining areas. Arrowheads indicate double positive cells. Scale bar, 100 µm. Nuclei were counterstained DAPI.

To delineate the downstream lineage of Cd63^+^ cells, we captured TdTomato^+^ liver cells from *CD63CreERT2*; *Rosa26‐TdTomato* mice by FACS and applied SMART2‐seq, which is more sensitive than 10x genomic method in terms of the number of genes detected and the average read number for each gene. All 63 qualified single cells were annotated as hepatocytes or BECs, based on previous identified signatures of hepatocytes and BECs in single‐cell transcriptome data (**Figure** **6**A,B). Trajectory analysis show that these cells form three branches. One branch is representative of hepatocytes composed of Group 1 cells; the other two branches are BECs contributed by both Group 2 and 3 cells (Figure 6C,D).

We further characterized the three cell groups by the genes that are expressed in each group (Table S1, Supporting Information). A number of classic signature genes of hepatocytes and BECs were identified (Figure 6E). We calculated a score for each group that reflects the expression of the hepatocyte‐specific *Cyp* genes and found that the Cyp score was the highest in Group 1. BECs/LSCs markers *Sox17* and *Cd24a* were highly expressed in Group 2. *Krt19* was also significantly expressed in Group 2 (Figure 6E). GO terms related to metabolic pathways were enriched in genes expressed in Group 1, indicating that Group 1 cells are hepatocytes (Table S2, Supporting Information). Group 2 and 3 both expressed *Cd63*, and GO terms related to stem cells were enriched in both Group 2 and 3 but not in Group 1, indicating that both Group 2 and 3 might be stem cells but with different properties (Figure 6E,F). 2870 genes were differentially expressed between Group 2 and 3 (Table S3, Supporting Information). GO terms related to stem cell differentiation and regulation of stem cell proliferation were enriched in Group 2 while those related to negative regulation of stem cell differentiation were enriched only in Group 3 (Figure 6F; Table S4, Supporting Information). These findings strongly suggest that Group 2 are activated liver stem cells (ALSCs) while Group 3 are quiescent liver stem cells (QLSCs). In those genes differentially expressed genes between Group 2 and 3, the surface antigen *Ncam1* (*CD56*) was exclusively expressed in Group 2 (Figure 6E), suggesting that the CD56 may be a surface marker for ALSCs.

NCAM1 is already known as a definitive NK cell marker.^[^ ^23^, ^24^ ^]^ To confirm which cell group that NCAM1 could mark in the injured liver, further analysis was performed. TSNE plots of Cd63‐lineage cells indicated that Ncam1 was mainly expressed in the ALSCs group, which was negative for hematopoietic markers: Cd45, Cd163 or Cd8 (Figure 6G). Immunostaining results demonstrated that NCAM1 marked a sub‐population of KRT19^+^ BECs in the portal vein area. CD45 (pan‐hematopoietic marker) and CD163 (macrophage marker) were expressed around BECs, but not on BECs (Figure 6H,I). However, in the liver parenchyma around central vein, NCAM1 and CD45/CD163 double positive cells could be detected, which represented NK cells, macrophages, *etc*.

These results indicated that liver stem cells comprised quiescent and active states, which could be distinguished by CD56.

### 2.4 CD63^+^CD56^−^ QLSCs Could be Activated by VEGF‐A and bFGF

**Figure 7:**
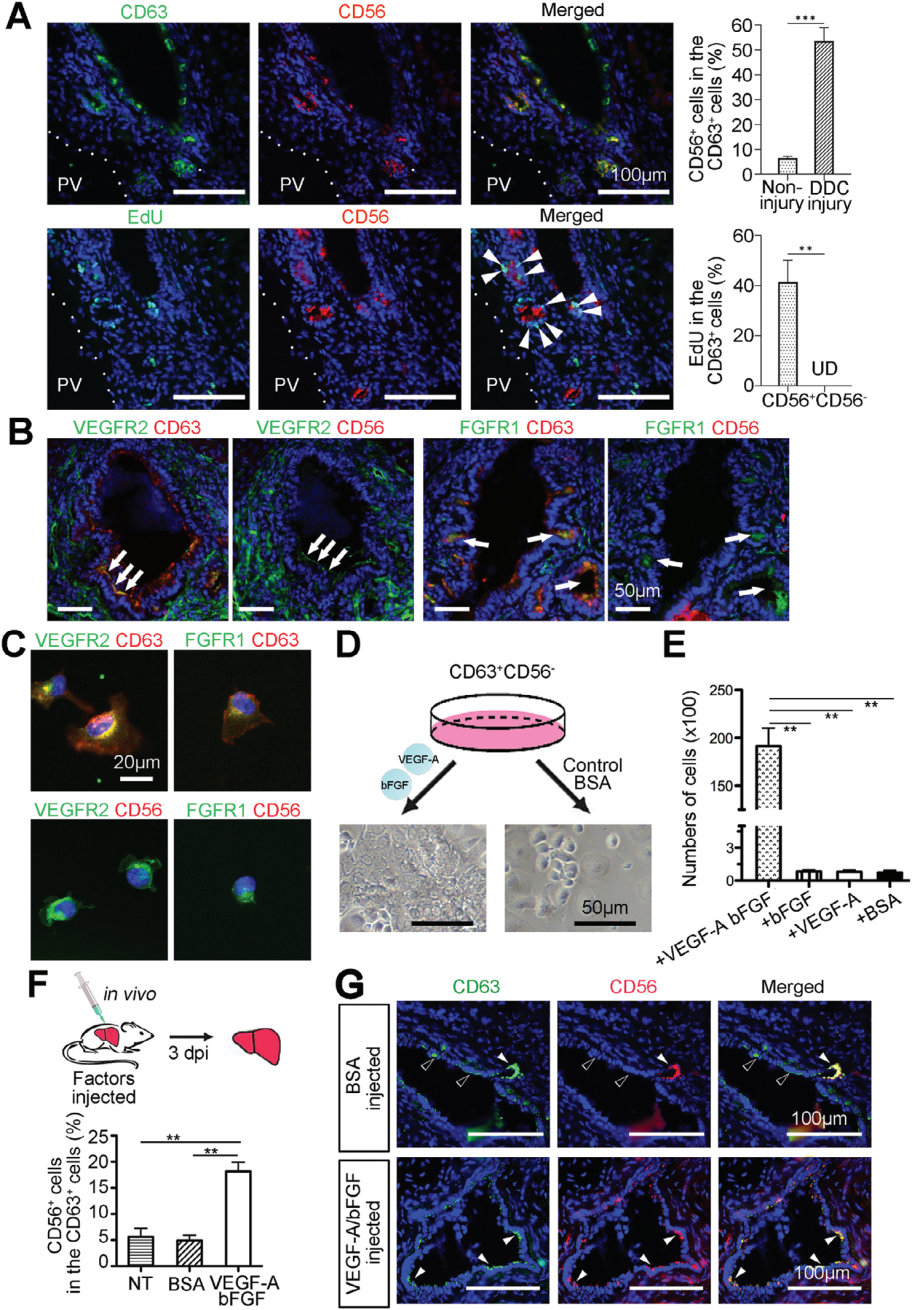
Activation of quiescent liver stem cells (QLSCs) by VEGF and bFGF. A) Representative immunostaining images showed co‐expression of CD63 and CD56, or EdU and CD56 on adjacent serial sections, indicating that CD56^+^CD63^+^ ALSCs are mitotically active. Arrowheads indicated proliferated ALSCs. Scale bar, 100 µm. Nuclei were counterstained DAPI. Data were shown as mean ± SD. n = 3. ^**^ *p* < 0.01. ^***^ *p* < 0.001. UD, undetected. B) Representative immunostaining images showed expression of VEGFR2/CD63/CD56 or FGFR1/CD63/CD56 in situ. Arrows indicated that CD56^−^CD63^+^ QLSCs expressed VEGFR2 and FGFR1. Scale bar, 50 µm. C) Representative immunostaining images showed isolated CD56^−^CD63^+^ cells expressed VEGFR2 and FGFR1 in vitro. Scale bar, 20 µm. D) Schematic diagram of CD56^−^CD63^+^ QLSCs that were treated with VEGF‐A and bFGF, or treated with solvent (bovine serum albumin, BSA). Phase contrast images showed cell expansion in the presence of VEGF‐A/bFGF but not in the control group. E) Quantification of cell numbers in each group, which CD56^−^CD63^+^ cells grown in medium added with VEGF‐A and bFGF, each factor alone, or BSA, respectively. Synergistic effect of VEGF‐A and bFGF were indicated. Results are represented as mean ± SD. *n* = 3. ^**^ *p* < 0.001. F,G) CD56^−^CD63^+^ QLSCs were activated in vivo. F) Mice were directly injected with VEGF‐A and bFGF into a liver lobule and were analyzed 3 days post‐injection. Quantification of CD63^+^CD56^+^ cells from VEGF‐A/bFGF injection compared to non‐treated (NT) liver and BSA‐treated livers. Data were shown as mean ± SD. *n* = 3. ^**^ *p* < 0.001. G) Representative images showed CD63^+^CD56^+^ cells in both BSA‐ and VEGF‐A/bFGF‐ treated livers 3 days post injection. Hollow arrowheads indicated CD63^+^CD56^−^ cells while white arrowheads indicated CD63^+^CD56^+^ cells, noticing increasing numbers of CD63^+^CD56^+^ cells. Scale bar, 100 µm.

To investigate whether CD56 could label ALSCs, double staining of CD56 and CD63 were further applied. In non‐injury livers, CD63^+^CD56^+^ cells accounted for 6.48% ± 0.45% of CD63^+^ cells and the number rose to 53.51% ± 3.16% in DDC injury liver (**Figure** **7**A). Our work suggested that QLSCs could be triggered to active state upon injury. The proliferation ability of CD63^+^CD56^+^ ALSC was evaluated by EdU incorporation. Results indicated that 41.38% ± 5.09% CD63^+^CD56^+^ cells were EdU positive while CD63^+^CD56^−^ cells were EdU negative (Figure 7A). When CD63^+^CD56^−^ and CD63^+^CD56^+^ populations from the DDC injury liver were isolated by FACS (Figure S6A, Supporting Information) and cultured under the same conditions, these two cell populations showed remarkably different characteristics. The CD63^+^CD56^+^ cells generated long‐lived cultures (Figure S6B2, Supporting Information), while CD63^+^CD56^−^ cells did not expand (Figure S6B1, Supporting Information). We conclude that within the CD63^+^ pool in the liver, CD56 marked ALSCs, while QLSCs were negative for CD56.

Previously study suggested the VEGF and FGF signal pathways were involved in the activation and expansion of quiescent neural stem cells.^[^ ^25^ ^]^ Therefore, in our own data, we investigated whether VEGF and FGF signaling pathways are involved in the activation and expansion of resting hepatic stem cells. By GO enrichment analysis of group specific genes, we observed VEGF and FGF signaling pathways in the genes specifically expressed in group 2 (ALSCs) (Table S4, Supporting Information), which indicated the roles of VEGF and FGF signaling pathway in the activation and expansion of LSCs. To verify whether CD63^+^CD56^−^ QLSCs could be activated by specific pathways, further studies were performed. The key signaling receptors were examined. VEGFR2 and FGFR1 were found to be expressed by CD63^+^CD56^−^ QLSCs (Figure 7B,C). Screening combination of growth factors (VEGF and FGF family) were examined for activation of CD63^+^CD56^−^ cells. As described above, CD63^+^CD56^−^ QLSCs did not expand well in culture. However, in the presence of both bFGF and VEGF‐A in ScmA medium, cells proliferated vigorously (Figure 7D,E). Other factors or one of the two factors could not promote growth of cells. For in vivo studies, VEGF‐A and bFGF were injected directly into a liver lobule, and the injected livers were examined 3 days after injection (Figure 7F). In control animals injected with BSA, few CD63^+^CD56^+^ ALSCs were detected (5.61% ± 2.3%, Figure 7F,G). In experimental animals injected with bFGF and VEGF‐A, increased numbers of CD63^+^CD56^+^ cells (18.15% ± 3.0%) were detected, consistent with the notion that bFGF and VEGF‐A together can activate QLSCs and/or expand ALSCs in vivo. These results indicated that CD63^+^CD56^−^ QLSCs could be induced by bFGF and VEGF‐A to an active state.

**Figure 8:**
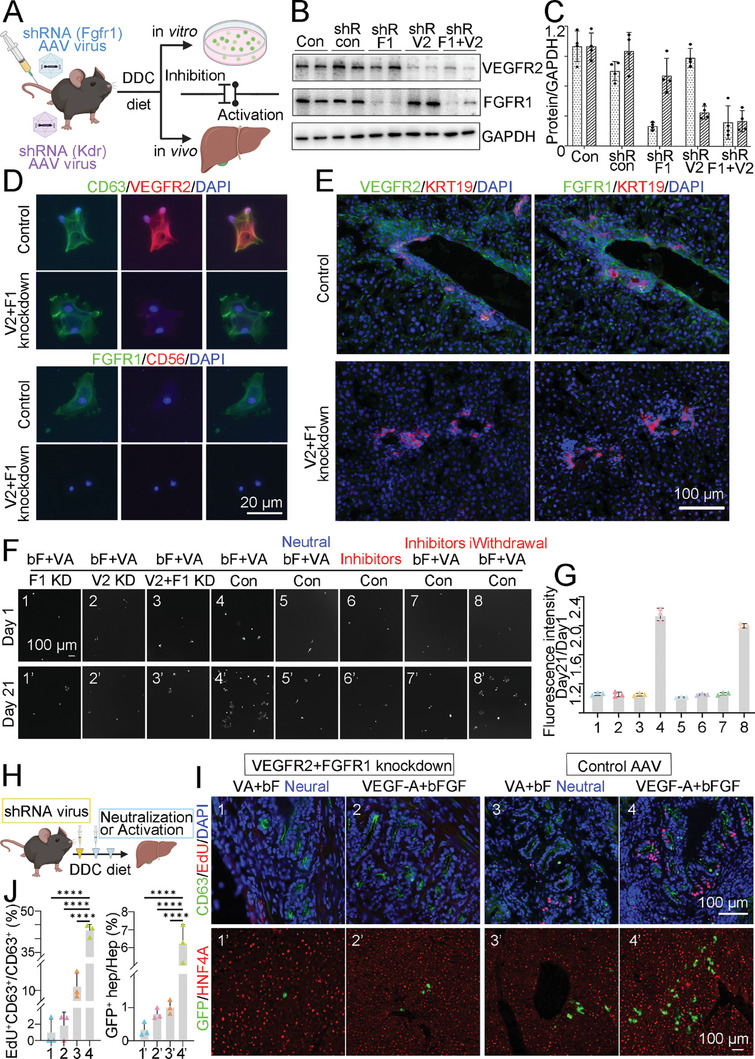
VEGF and FGF signaling regulates proliferation and differentiation of QLSCs. A) Schematic diagram of experiments after silencing expression of Vegfr2 (Kdr) and Fgfr1. B,C) Western blotting and quantifications of liver lysates from each experimental group. FGFR1 or VEGFR2 protein pression was compared with GAPDH. Results are represented as mean ± SEM. *n* = 4. shR con, shRNA AAV control group. shR F1, shRNA Fgfr1 AAV group. shR V2, shRNA Vegfr2 AAV group. shR F1+V2, shRNA Fgfr1 and Vegfr2 AAV group. Con, control. D,E) Immunostaining for CD63, CD56, KRT19, VEGFR2 and FGFR1 in CD63^+^CD56^−^ cells or liver section, isolated from shRNA (Vegfr2‐Fgfr1) AAV mice or control mice. Scale bar, 20 µm (D) or 100 µm (E). Nuclei were counterstained DAPI. F) Isolated QLSCs stained with Vybrant CFDA SE were imaged by fluorescence microscope. Day 1, up panels (1–8) showed cells cultured for 1 day. Day 21, bottom panels (1′‐8′ corresponding to 1–8) showed cells cultured for 3 weeks. F1 KD, Fgfr1 knockdown. V2 KD, Vegfr2 knockdown. V2+F1 KD, Vegfr2 and Fgfr1 both knockdown. Con, control AAV group. bF+VA, addition of bFGF and VEGF‐A in the culture medium. Neutral, neutralization of bFGF and VEGF‐A by antibodies (Mouse VEGF164 Antibody, 150 ng mL^−1^ and FGF basic/FGF2/bFGF Antibody, 200 ng mL^−1^). Inhibitors, addition small molecule inhibitors of VEGFR2 and FGFR1 (PD166866, 10 µm and Anlotinib, 10 µm). iWithdrawal, withdrawal of chemical inhibitors. G) Fluorescence intensity of each group read by Microplate Reader. Results were showed as value ratio (D21/D1). Results are represented as mean ± SEM. *n* = 3. H–J) Proliferation and differentiation ability of CD63^+^ cells influenced by VEGF‐FGF signaling in vivo. H) Schematic diagram of AAV injection, injury process and interfering strategy. I) Representative images of immunostaining for CD63, EdU, HNF4A and GFP in the liver. Panel 1–4, EdU staining showed proliferation CD63^+^ cells. Panel 1′‐4′, GFP marked differentiated hepatocytes from CD63 lineage‐tracing mice liver. VA+bF Neural, neutralization of bFGF and VEGF‐A by antibodies. VEGF‐A+bFGF, VEGF‐A and bFGF injected group. Scale bar, 100 µm. Nuclei were counterstained DAPI. J) Calculation of EdU^+^CD63^+^/total CD63^+^ cells (left) and GFP^+^ hepatocytes/total hepatocytes (right) in each group. Data presented as mean ± SEM. *n* = 3. ^****^ *p* < 0.0001.

To further investigate the role VEGF and FGF signaling during injury repair, we silenced VEGFR2 (Kdr) and FGFR1 expression in the liver by adeno‐associated virus (**Figure** **8**). Both of the shRNA(Kdr) and shRNA(Fgfr1) AAV were generated and injected into mice for further examination. Expression of VEGFR2 and FGFR1 were efficiently silenced no matter in the liver or isolated CD63^+^CD56^−^ QLSCs (Figure 8A–E). According to the above results, VEGFR2 and FGR1 were equally important, and loss one of them could not promote QLSCs to proliferating. Neutralization of VEGF‐A and bFGF or addition chemical inhibitors of VEGFR2 and FGFR1 could repress the growth ability of LSCs. This could be rescued after withdrawal of inhibitors and LSCs went to expansion again (Figure 8F). All the groups were detected by CFDA SE Cell Tracer and calculated with fluorescence intensity (Figure 8G). After confirming both of the two signaling were required for LSCs, *CD63CreERT2*; *Rosa26R‐GFP* mice, were applied for tracing cell fate upon injury (Figure 8H–J). Results suggested that by silenced expression of VEGFR2 and FGFR1, VEGF‐A and bFGF were not able to provoke LSCs into proliferation or differentiation (Figure 8I,J). Further, neutralization of VEGF‐A and bFGF could significantly block activation of LSCs. It is noteworthy that, these two factors could augment proliferation and contribution of LSCs during injury process.

These data strongly suggested VEGF and FGF signaling play an irreplaceable role on activation of LSCs under certain injury circumstance.

## 3 Discussion

In this study, via single‐cell transcriptome analyses, we identified biliary epithelium cells and their subpopulation, which including quiescent and activated liver stem cells. Most importantly, we identified CD63 as a bona fide novel LSCs cell surface marker, which labeled both QLSCs and ALSCs. Previously, several distinct cell types have been proposed as putative stem cells in the liver. Those include FoxL1^+^, OPN^+^, Sox9^+^, Lgr5^+^ or Hnf1b^+^ positive cells originated from the bile duct.^[^ ^1^, ^16^, ^18^ ^]^ From our analyses, CD63 labels LSCs more specifically than any of the previously proposed/used putative LSCs markers. The locations of CD63 LSCs are concentrated in the portal triad area, specifically CoH and PBG. Lineage tracing experiments clearly demonstrated that CD63^+^ LSCs responded to liver injury and produced a significantly cells to repair both hepatic and cholangiocytic lineages. This bipotent ability to repopulate cells of both lineages unequivocally implicate stem cell characteristics.

The adult liver is superbly able to self‐maintain and regenerate, but the cells able to mediate liver regeneration, whether it is the LSCs population or hepatocytes, have been heavily debated.^[^ ^1^, ^2^, ^12^ ^]^ The claim that LSCs exist in the adult liver and may contribute significantly to organ renewal has been challenged with observations indicating that mature hepatocytes are endowed with progenitor‐like features during organ regeneration after injury.^[^ ^5^, ^6^ ^]^ In addition to merely replenishing the hepatocyte lineage, mature hepatocytes have also been shown to take on a bile duct cell‐like fate upon DDC injury or via genetic modifications, by turning on ductular specific genes such as Opn, Sox9, and Hnf1b.^[^ ^26^ ^]^ However, the transition from hepatocytes into ductular cells is somewhat incomplete, as these cells retain characteristics of hepatic progenitors that can easily revert back into hepatocytes. Moreover, despite expressing ductal markers, these hepatocyte‐derived cells do not reconstitute the typical bile duct structure and they do not participate in the portal bile duct formation. We, in this study, provide powerful new tools that could be used in the future to assess exact contributions of LSCs during liver injury repair by potential elimination of the CD63 lineage through mouse genetic manipulations. This new LSCs marker, CD63, will help clarify a number of contentious issues surrounding contributions of LSCs versus hepatocyte during various kinds of liver injury repair.

In non‐hepatic tissues, it has been reported that adult tissue stem cells exist in varying states of quiescence and activation.^[^ ^4^, ^27^ ^]^ The quiescent, non‐cycling state allows cells to withstand metabolic stress and persist over an animal's lifetime. Quiescent stem cells have low RNA content and lack cell proliferation markers.^[^ ^4^ ^]^ Earlier reports showed that CD56 (NCAM) marked a small portion of cells in the bile duct that may include stem cells.^[^ ^18^ ^]^ We extended these studies by showing that the adult LSCs could be segregated into active and quiescent populations based on CD56 expression. Single‐cell transcriptome analysis further revealed that while VEGF and MAPK signaling pathways might participate in the activation of quiescent LSCs. This was in according with our previous study, which proved that quiescent neural stem cells can activated by stimulus with FGF and VEGF.^[^ ^25^ ^]^

Specialized microenvironment, the so called stem cell niche,^[^ ^28^ ^]^ provides the correct combination of signals either through cell‐cell physical contact or secretion of soluble factors in a paracrine manner, keeping the homeostasis of adult tissue stem cells. Interestingly, the common theme about tissue quiescent stem cell activation appears to involve injury signals (e.g., cytokines) as well as vasculature reconstruction signals (e.g., VEGF), which are also abundant after injury. Whether normal tissue turns over and injury repair share common signaling for stem cell activation remain to be determined. Yet, it still worth mentioning that perhaps not all tissues manifest active tissue turnover under physiological condition, but most tissues initiate tissue repair after injury, therefore placing VEGF‐mediated QLSC activation at the center stage of tissue‐specific adult stem cell biology.^[^ ^25^ ^]^

CD63 is a broadly applied marker for identifying extracellular vesicles (EVs), which are well studied their effects of injury repair, inflammation or tumor pre‐metastasis in the liver.^[^ ^29^ ^]^ However, the relationship between EVs and LSCs is poorly understood. It is notified that CD63 expression of LSCs were elevated during injury process, and whether these CD63 proteins were assembling EVs is deserved further investigation. Also it is widely acknowledged EVs contain bioactive components: microRNAs, lipids, proteins *etc*.^[^ ^30^, ^31^ ^]^ This prompts us to explore crosstalk between LSCs‐derived EVs and injury microenvironment, which might be interfered with FGF/VEGF signaling pathway.

Our data not only provide a marker for identifying liver stem cells, but also shed light on mechanisms of activating QLSC. Further studies on promoting endogenous repair during liver injury will lead to deeply understanding about the ability and mechanism of orchestrating liver regeneration.

## 4 Experimental Section

### Mice Studies

All animals were kept in a Specific Pathogen Free animal facility and animal experiments were performed in agreement with the NIH Guide for the Care and Use of Laboratory Animals. All procedures and protocols were performed according to guidelines approved by Committee on Ethics of Medicine, Naval Medical University (NSFC31970753). Male and female mice were used and did not show sex bias differences. Rosa26‐GFP (Stock No: 007906) and Rosa26‐tdTomato (Stock No: 007914) were from the Jackson Laboratory. Krt19CreERT mice were from Dr. Guoqiang Gu's lab.^[^ ^32^ ^]^ Fah^−/−^Rag2^−/−^Il2rg^−/−^ mouse were kept as previously described.^[^ ^33^ ^]^ The CD63CreERT2 knock‐in mouse line was generated by knocking CreERT2‐pA element into the sites before the initiator ATG of the Cd63 gene (Exon2), using the CRISPR/Cas9 technology. The CD63CreERT2 knock‐in mouse line was generated by knocking CreERT2‐pA element into the sites before the initiator ATG of the Cd63 gene (Exon2), using the CRISPR/Cas9 technology. The sequence of knock‐in sites: ggcggggagctgcggatgtggcggcccctcggcctgctctacctgctctaatccttggtgttctccgcggccccag*GCCCAACAGCC*ATGGCGGTGGAAGGAGGAATGAAGTGTGTCAAGTTTTTGCTCTACGTTCTCCTGCTGGCCTTCTGC (upper case: Exon2; lower case: Intron1; underlined: PAM). 5′ homologous arm, CreERT2‐pA and 3′homologous arm were inserted into the donor vector. Cas9 mRNA, gRNA (5′‐AGGCCCAACAGCCATGGCGG TGG‐3′) and donor vector were injected into fertilized oocytes. Founders were identified by PCR‐amplification (Primers: 5′‐GGGACTCTATGTAGCCTTTGTTGA‐3′, 5′‐GGGCTGGGGGCTGATATTGATGTA‐3′; 5′‐GGGGCTGGGCTTCCTCTCG‐3′, 5′‐CTTGCAGAGGGCCCAGGTTTAGTT‐3′).

For labeling cells, Krt19CreERT or CD63CreERT2; Rosa26‐GFP/tdTomato mice at the age of 6–8 weeks were given 0.15 mg g^−1^ body weight of tamoxifen (Sigma–Aldrich). Acute CCl4 (Sigma–Aldrich) injury was performed by injecting 0.5 ul g^−1^ body weight. A diet containing 0.1% DDC (wt/wt) (Sigma–Aldrich) were put on mice. Fah^−/−^Rag2^−/−^Il2rg^−/−^ mouse were weaned from NTBC after transplanted with 1 × 10^6^ cells and sacrificed 8 weeks later. DDC‐injury mice were sacrificed 3 weeks after transplantation with 1 × 10^6^ cells.

### Liver Cell Isolation and FACS

Cell isolation was performed as previously described.^[^ ^34^ ^]^ Briefly, livers from adult mice were perfused with 0.5 mg mL^−1^ collagenase D (Roche) through the portal vein. After pelleting down the hepatocytes, the cells remaining in the supernatant were equilibrated with OptiPrep (Sigma–Aldrich) to 17% final concentration. After centrifuging at 400 g for 15 min, cells band at the interface were collected and red blood lysis buffer (Beyotime) were used after wash. Cell viability was at least 95% (Trypan blue exclusion, Sigma–Aldrich). Cells were filtered through a 40 µm sieve, stored in PBS with 2% FCS. FACS antibodies were incubated for 30 min at 4 °C. Cells were analyzed or sorted with BD flow cytometer (BD biosciences) using a 100 µm nozzle.

### Generation and Injection of Adeno‐Associated Virus

For silencing expression of Vegfr2 (Kdr) and Fgfr1, shRNA(Kdr)/shRNA(Fgfr1)/shRNA(Control)‐ adeno‐associated virus (AAV) was generated, respectively, by OBiO Technology (Shanghai). pAAV‐U6‐shRNA(X)‐CMV‐mScarlet‐WPRE was applied as AAV vector. The oligonucleotide sequences of shRNA were: 5′‐CCCGTATGCTTGTAAAGAA‐3′ (Kdr), 5′‐TATACGTGCTTGGCGGGTAACTCTA‐3′ (Fgfr1), 5′‐CCTAAGGTTAAGTCGCCCTCG‐3′ (Control). AAV at 5×10^7^ viral genomes per gram body weight (vg g^−1^) in 100 µL PBS was injected into each mouse via the tail vein and bile duct.

### Chemicals and Proteins Addition/Injection

For in vitro, small molecules PD‐166866 and Anlotinib (Selleck), or growth factors VEGF‐A and bFGF, or neutral antibodies VEGF164 Antibody and bFGF Antibody, were dissolved and added in the medium. For in vivo, factors or neutral antibodies of VEGF‐A and bFGF, injected into the liver lobule and portal vein.

### Cell Culture and Differentiation

Sorted cells were cultured in ScmA medium,^[^ ^22^ ^]^ supplemented with 10% fetal bovine serum (HyClone), 1% penicillin/streptomycin, 0.1 mmol L^−1^ 2‐mercaptoethanol (both from GIBCO), 10 ng mL^−1^ HGF, 10 ng mL^−1^ EGF (both from R&D Systems), insulin‐transferrin‐selenium (ITS), 10–7 mol L^−1^ dexamethasone, 10 ng mL^−1^ nicotinamide, and 50 mg mL^−1^ gentamicin (all from Sigma–Aldrich) in DMEM/F12 (Gibco). For the first 4 days after sorting, culture medium was supplemented with 10 µm Y27632 (Sigma). Both hepatic and cholangioctic induction, PAS staining and Indocyanin Green metabolism were performed according to published methods.^[^ ^19^ ^]^ For activating CD63^+^CD56^−^ cells, the cells were collected with culture medium, and 50 cells were seeded per well in a 96‐well/plate. For clonogenic assays, single‐cell suspensions were sorted and directedly seeded in 96‐well plates at a ratio of 1 cell per well. Cells were cultured as described above.

### RNA Isolation, RT‐PCR, and Quantitative Q‐RT‐PCR

Total RNA from cells was isolated with Trizol reagent (Invitrogen). Reverse‐transcriptase reactions were carried out with SuperScript II reverse transcriptase (Invitrogen) according to the manufacturer's protocol. Q‐RT‐PCR was performed in three repeats of each sample with ABI‐7900 (Applied Biosystems) by SYBR Green master mix (Applied Biosystems). mRNA abundance was determined by normalization of data to the expression level of GAPDH mRNA. mRNA expression in normal liver was taken as the baseline and considered equal to 1.

### Immunohistochemistry, Immunofluorescent Staining and Immunostaining

Liver tissues were fixed in 4% paraformaldehyde (Sigma–Aldrich) and embedded in OCT (Sakura) or paraffin. Where appropriate, sections were permeabilized with 0.3% Triton‐X100 (Sigma–Aldrich) and blocked with 1% bovine serum albumin (Sigma–Aldrich). For immunohistochemistry, quenching of endogenous peroxidase was performed prior to antibody incubation. After incubation with primary antibodies overnight at 4 °C, sections were washed and incubated with secondary antibodies conjugated with fluorescent dye or HRP at 37 °C for 30 min. DAB (Maixin) staining was applied on the sections of incubated with HRP‐conjugated antibody. For immunostaining, cells were fixed in 4% paraformaldehyde and blocked with 1% bovine serum albumin solution containing 0.3% Triton‐X 100. All primary antibodies were incubated overnight at 4 °C and secondary antibodies at 37 °C for 30 min. Slides were mounted with coverslips using mounting medium with DAPI (Southern Biotech). Images were acquired by Nikon microscope and ImageJ software was used for quantification. Five random fields from each liver lobule section were processed for quantification. Each image was representative of at least three individual mice samples.^[^ ^35^ ^]^

### Live Cell Staining and Tracing

Cultured live cells were stained with Vybrant CFDA SE Cell Tracer Kit (Invitrogen) according to the manuals. Samples were imaged with fluorescence microscope (Nikon). For quantification, they were read by Microplate Reader (TECAN) and recorded.

### Western Blotting

Tissue samples were harvested and lysis in RIPA buffer (Thermo), 1 mm PMSF (Thermo), Protease and phosphatase inhibitor cocktail (Thermo). The protein concentration was determined by Pierce BCA Protein Assay Kits (Thermo). An equal amount of protein extracts (10 µg) mixed with loading buffer (Beyotime) were subjected to 10% SDS‐PAGE and transferred to a 0.45 µm polyvinylidene fluoride membranes (Millipore), followed by blocking with 5% Non‐Fat Powdered Milk/TBST at room temperature for 1 h and incubation overnight with primary antibodies at 4 °C. Then membranes were washed and incubated at room temperature for 1 h with secondary antibodies. Finally, membranes were visualized using the enhanced ECL system (Thermo). The band intensity was quantified using ImageJ software.

### Sing‐Cell RNA‐Seq

The cell suspension was prepared as above and then diluted to a final concentration of 1 × 10^6^ mL^−1^ in PBS. The volume of single‐cell suspension that was required to generate 10 000 single‐cell GEMs (gel beads in the emulsion) per sample was loaded onto the Chromium Controller (10X Genomics). Libraries were prepared using the Chromium v3 Single Cell 3′ Library and Gel Bead Kit (10X Genomics) according to the manufacturer's specifications. Final library quantification and quality control were performed using a DNA 1000 chip (Agilent Technologies), followed by sequencing on Illumina NovaSeq 6000. Raw sequence data were aligned to the mm10 (Ensembl 84) reference genome, and cell numbers along with unique molecular identifiers (UMIs) were estimated, using the CellRanger (version 3.1.0), the single‐cell software suite from 10X Genomics. Downstream analyses were performed using the Seurat R package (version 3.1.5).

### Data filtration and Data Analysis

Low‐quality cells elimination was performed according to previous strategies. Cell libraries with low complexity (fewer than 200 expressed genes) were excluded. Cells with mitochondrial gene‐expression fractions greater than thresholds for each sample were excluded. The thresholds were determined by considering a median‐centered median absolute deviation‐variance normal distribution; cells with mitochondrial read fraction outside of the upper end of this distribution were excluded (where outside corresponds to *p* < 0.05; Benjamini–Hochberg‐corrected). For single‐cell RNA sequencing analysis, the LogNormalize method with a scale factor of 10 000 was used for normalization. The top 5000 variable features were extracted using the FindVariable Features function. The data were scaled according to the mitochondrial percentage using the ScaleData function. The clustering results were visualized using t‐SNE and UMAP plots. Biological identity of each cells was annotated by AddModuleScore function with cell type features that identified by R package scmap with using of a human liver single‐cell data (GSE124395). The function FindAllMarkers was used to find the DEGs in each cluster. The R package clusterProfiler was applied to perform GO enrichment analysis. Single‐cell pseudotime trajectories were constructed with MONOCLE (version 2.18).

### Statistical Analysis

The data were obtained through independent experiments or repeated measurements, with sample sizes of n = 3 or 4, respectively. For experimental data analysis, statistical analysis was performed with GraphPad Prism 9.4. Mean values between the two groups was compared by performing Student's *t*‐test. Multiple group comparisons were performed using the one‐way ANOVA or two‐way ANOVA. The data were expressed as mean ± SD/SEM. ^*^ *p* < 0.05; ^**^ *p* < 0.01; ^***^ *p* < 0.001, ^****^ *p* < 0.0001 were considered statistically significant. ns, not significant.

Detailed information of antibodies, regents and primers were listed in Supporting Information.

## Conflict of Interest

The authors declare no conflict of interest.

## Supporting information

Supporting Information

## Data Availability

The data that support the findings of this study are available from the corresponding author upon reasonable request.
