# Supplementary material for: VEGF‐FGF Signaling Activates Quiescent CD63+ Liver Stem Cells to Proliferate and Differentiate
Source: Adv Sci (Weinh). 2024 Jun 17;11(33):2308711. doi: 10.1002/advs.202308711 (PMC11434209; doi:10.1002/advs.202308711)
Supplement: Supplementary file 1 — Supporting Information [file ADVS-11-2308711-s001.pdf]

ADVANCED SCIENCE
Open Access

Supporting Information

for *Adv. Sci.*, DOI 10.1002/advs.202308711

VEGF-FGF Signaling Activates Quiescent CD63^{+} Liver Stem Cells to Proliferate and Differentiate

Fei Chen, Kunshan Zhang, Minjun Wang, Zhiying He, Bing Yu, Xin Wang, Xinghua Pan, Yuping Luo, Shoujia Xu, Joseph T.Y. Lau, Chunsheng Han, Yufang Shi, Yi E. Sun, Siguang Li and Yi-Ping Hu**

Supporting Information

VEGF-FGF Signaling Activates Quiescent CD63⁺ Liver Stem Cells to Proliferate and Differentiate

Fei Chen, Kunshan Zhang, Minjun Wang, Zhiying He, Bing Yu, Xin Wang, Xinghua Pan, Yuping Luo, Shoujia Xu, Joseph T.Y. Lau, Chunsheng Han, Yufang Shi, Yi E. Sun, Siguang Li, Yi-Ping Hu**

These authors contributed equally: Fei Chen, Kunshan Zhang, Minjun Wang

Correspondence to: yphu9@yeah.net; lisiguang@tongji.edu.cn

This file includes:

Figures S1 to S6……………………………………………………………….…………….2

Tables of regents and primers…………………………………………………………...10

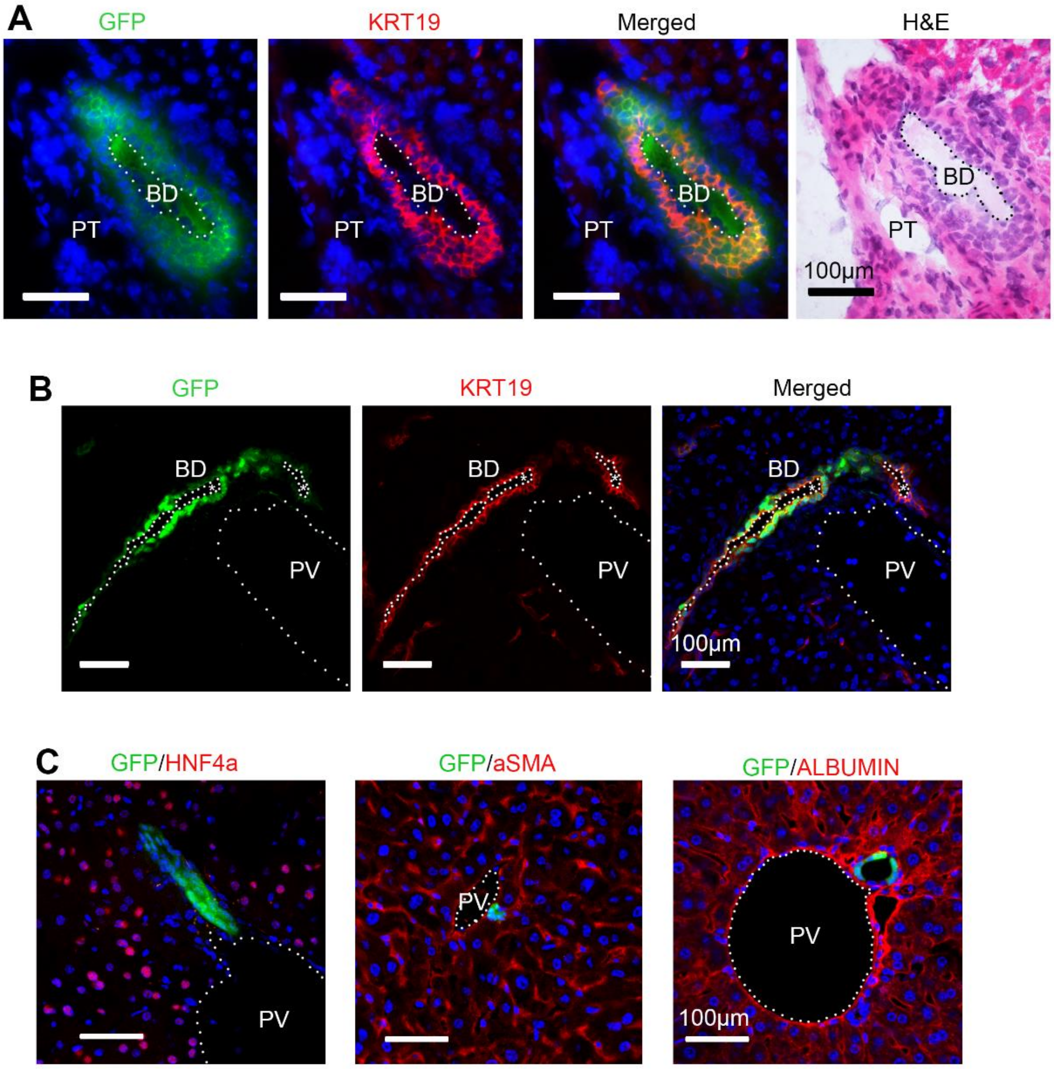

Figure S1. GFP^{+} cells expression analysis in the Krt19CreERT; Rosa26R-GFP mouse liver. A) Direct fluorescence of GFP combined with immunostaining for Krt19 and hematoxylin& eosin (H&E) staining shows GFP expression was restricted in the PT area 2 days after TM injection. B) Lower magnified view shows efficiency of GFP-labeled bile duct cell. C) Direct fluorescence of GFP combined with immunostaining for Hnf4a, aSMA and Albumin. Note that GFP-positive cells are specifically located in the portal area 2 days after TM injection.

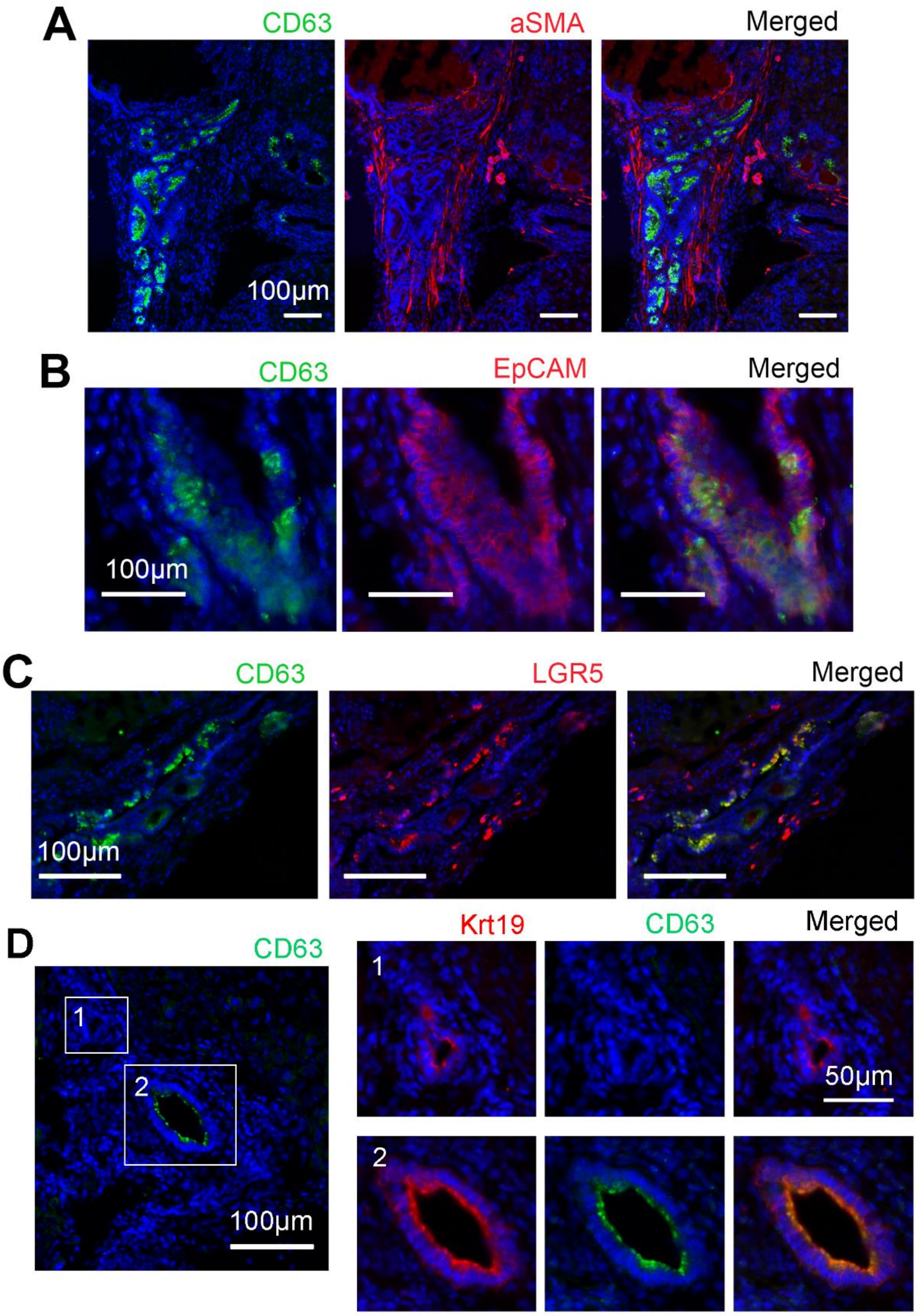

Figure S2. CD63⁺ cells co-stained with other markers in DDC injury mice and CD63⁺ cells in the CCl4 model. A) Double immunofluorescence staining of CD63 (green) and aSMA (red) showed PBG is surrounded by stromal cells. B) Epcam (red) was expressing all over the bile duct while CD63 (green) was expressing on the part of the duct. C) Staining of Lgr5 shows the restricted area of Lgr5 and CD63 co-expressing. D) Staining of CD63 and Krt19 in the CCl_4 injury model.

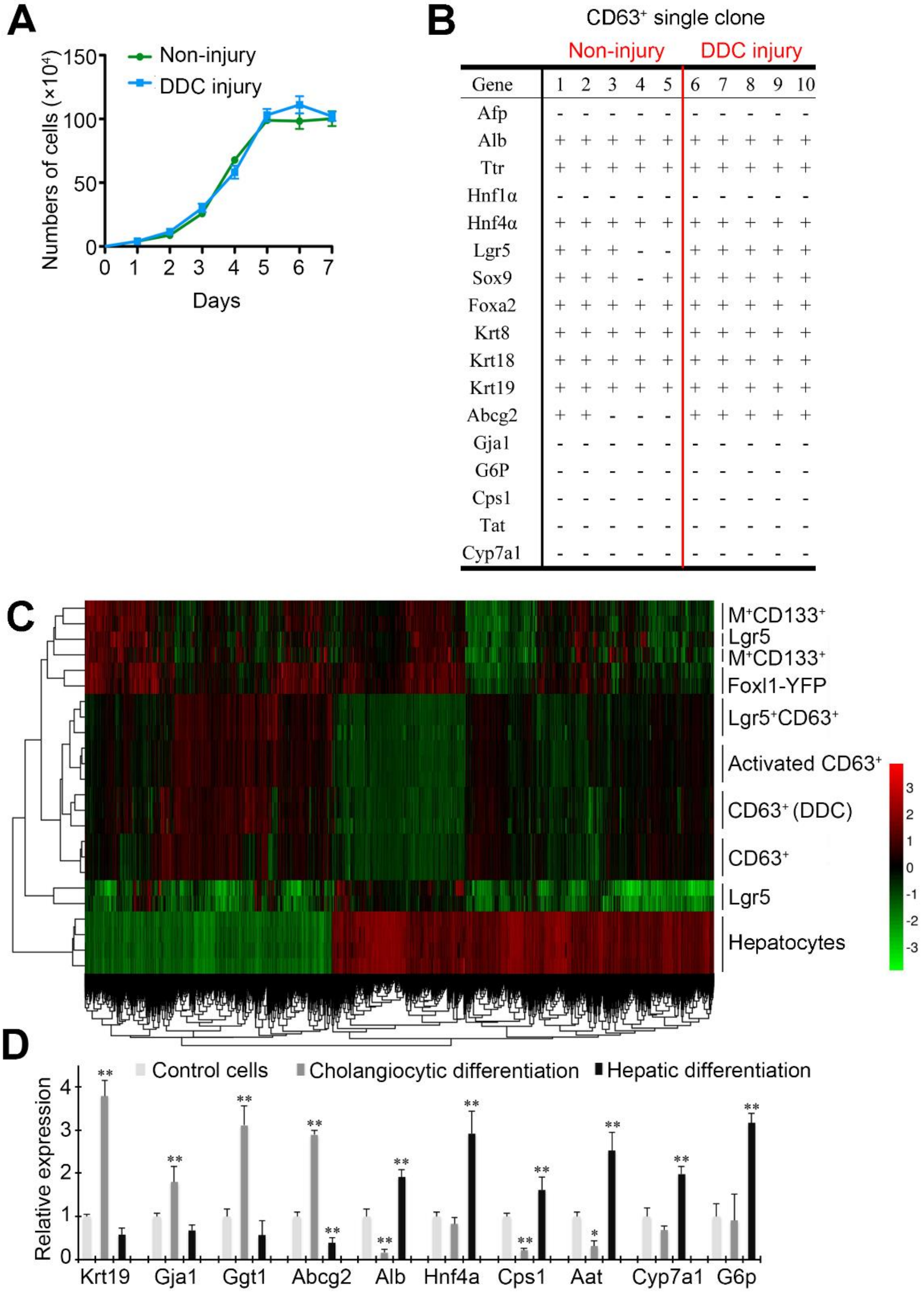

Gene	1	2	3	4	5	6	7	8	9	10
Afp	-	-	-	-	-	-	-	-	-	-
Alb	+	+	+	+	+	+	+	+	+	+
Ttr	+	+	+	+	+	+	+	+	+	+
Hnf1α	-	-	-	-	-	-	-	-	-	-
Hnf4α	+	+	+	+	+	+	+	+	+	+
Lgr5	+	+	+	-	-	+	+	+	+	+
Sox9	+	+	+	-	+	+	+	+	+	+
Foxa2	+	+	+	+	+	+	+	+	+	+
Krt8	+	+	+	+	+	+	+	+	+	+
Krt18	+	+	+	+	+	+	+	+	+	+
Krt19	+	+	+	+	+	+	+	+	+	+
Abcg2	+	+	-	-	-	+	+	+	+	+
Gja1	-	-	-	-	-	-	-	-	-	-
G6P	-	-	-	-	-	-	-	-	-	-
Cps1	-	-	-	-	-	-	-	-	-	-
Tat	-	-	-	-	-	-	-	-	-	-
Cyp7a1	-	-	-	-	-	-	-	-	-	-

Figure S3. CD63⁺ cells were self-renewing. A) Representative growth curves of different clones (non-injury, DDC injury) indicated typical sigmoid curve. B) Summary of gene expression in clones expanded from CD63⁺ cells. 5 different clones from WT and DDC respectively at passage 5 were analyzed by RT-PCR. Every gene symbols were listed left. Negative sign (-) indicates no expression while positive sign (+) indicates expression. PCR cycles: 35. C) Comprehensive transcriptome analysis of the CD63⁺ cells were displayed on heat map showing gene and sample clustering. CD63⁺ cells were from both non-injury and DDC injury mice. Lgr5⁺CD63⁺, expanded colonies from Lgr5⁺CD63⁺ cells. Activated CD63⁺ cells, activation of quiescent CD63⁺ cells. Foxl1-YFP are previously published data of liver stem cells. D) Quantitative polymerase chain reaction (Q-PCR) analysis revealed dynamic gene expressions during hepatic and cholangiocytic induction. Expression of Hnf4a, Cps1, Aat, Cyp7a1 and G6p represents hepatic maturation. Expression of Krt19, Gja1, Ggt1

and Abcg2 represent cholangiocytic induction. The results were relative expression calculated by normalizing to those un-induced cells. Data represent mean ± standard deviation, n=3. Control cells were $CD63^+$ cells cultured in ScmA medium. Data are the mean ± SD. $n = 3$. $^{**}p < 0.01$. $^{*}p < 0.05$.

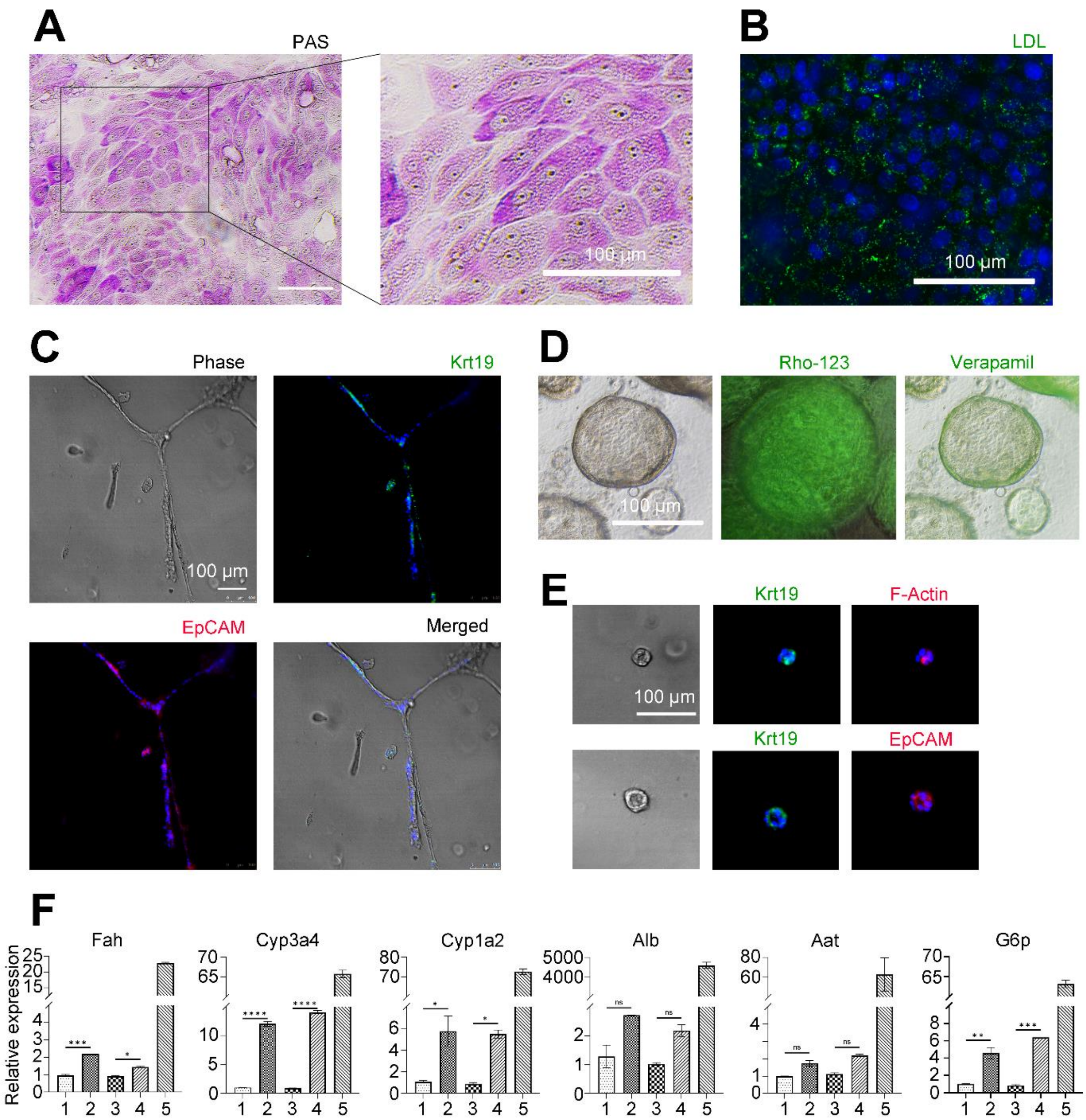

G

Sample	Fah Average Ct	Fah ΔCt	Cyp3a4 Average Ct	Cyp3a4 ΔCt	Cyp1a2 Average Ct	Cyp1a2 ΔCt	Alb Average Ct	Alb ΔCt	Aat Average Ct	Aat ΔCt	G6p Average Ct	G6p ΔCt
1	23.6	9.13	24.135	9.665	34.095	19.625	29.87	15.4	18.905	4.435	29.98	15.51
2	22.39	7.935	20.545	6.09	31.695	17.24	28.735	14.28	18.075	3.62	27.17	12.715
3	23.555	9.19	24.25	9.885	34.285	19.92	30.055	15.69	18.625	4.26	30.13	15.765
4	22.845	8.55	20.16	5.865	31.58	17.285	28.895	14.6	17.58	3.285	27.815	13.52
5	20.54	4.565	19.615	3.64	29.53	13.555	16.205	0.23	14.445	-1.53	25.545	9.57

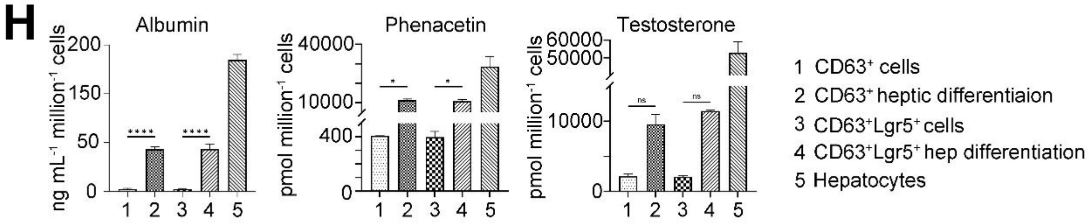

Figure S4. Differentiation of CD63$^+$Lgr5$^+$ cells. A-E) CD63$^+$Lgr5$^+$ cells were cultured in hepatic- and cholangiocytic- medium, respectively. A) PAS staining displayed mature hepatocyte character. Box inserted was high magnified view. B) Differentiated cells could take up fluorescent-labeled substrate of Dil-ac-low-density lipoprotein (Dil-Ac-LDL). C) Typical branching structures were

observed and stained with Krt19 or EpCAM. D) Cells were cultured in 3-dimensional condition. Transport of rhodamine 123 (Rho-123) into the central lumen of a cyst and verapamil (Ver) blocking Rho-123 transport. E) Typical morphology and staining of the cysts by Krt19, F-actin, Epcam). Nuclei were counterstained with Dapi. Scale bars, 100 µm. F-G) Quantitative polymerase chain reaction analysis revealed dynamic gene expressions of hepatic differentiation. Fah,Cyp3a4, Cyp1a2, Alb, Aat and G6p represents typical hepatic markers. The results were relative expression calculated by normalizing to those un-induced cells. Panel G shows original Ct value of each gene. Data represent mean ± standard deviation. *n* = 3. H) Functional assessment of Albumin secretion and drug metabolism. ELISA showed the levels of Albumin secretion. The metabolic products of phenacetin and testosterone were determined by liquid chromatography-tandem mass spectrometry according to standard curve. The data were represented as mean ± SD. *n* = 3. ****$p<0.0001$. ***$p<0.001$. **$p<0.01$.*$p<0.05$. ns, not significant.

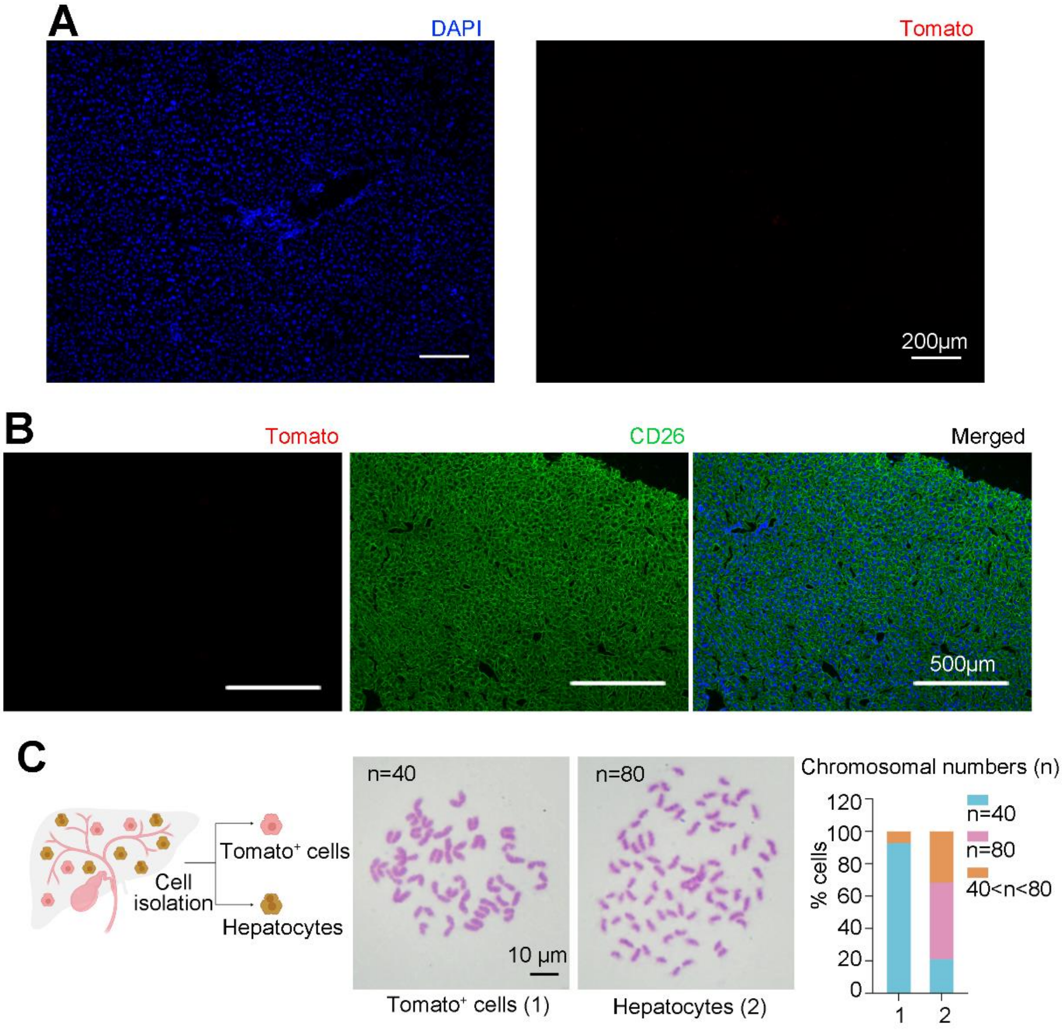

Figure S5. Characterization of CD63CreERT2;Rosa26-TdTomato mice. A) Representative images of CD63CreERT2;Rosa26-TdTomato mice without TAM treated. No fluorescence was detected. Scale bar, 200um. B) Lower magnified images of CD63CreERT2;Rosa26-TdTomato mice with TAM treated (0.15mg g^{-1} body weight). Scale bar, 500 µm. C) Chromosomal numbers were counted in both Cd63-lineage cells and hepatocytes. Representative karyotype of tomato^{+} cells and hepatocytes. Graph plot is the % of cells with chromosomal counts. n=40, n=80, aneuploidy 40<n>80. Scale bar, 10 µm.

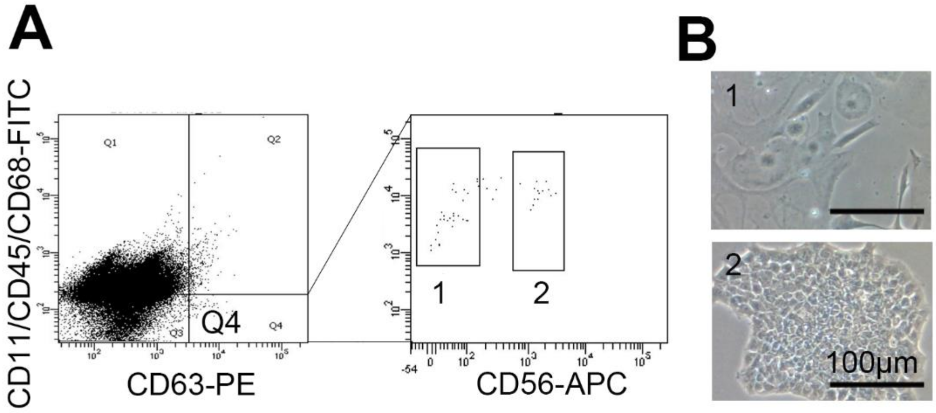

Figure S6. Growth state of CD56$^-$CD63$^+$ and CD56$^+$CD63$^+$ cells *in vitro*. A) Representative FACS plots showed isolation of CD56$^-$CD63$^+$ or CD56$^+$CD63$^+$ cells (both of which were from CD11$^-$CD45$^-$CD68$^-$ population) from DDC injury livers. 1, indicating CD56$^-$CD63$^+$ population. 2, indicating CD56$^+$CD63$^+$ population. B) Representative phase contrast images showed CD56$^-$CD63$^+$ cells (1) did not grow well, while CD56$^+$CD63$^+$ (2) could form large clones after culturing. Scale bar, 100μm.

Table S1 List of antibodies

Antibodies	Source	Cat. No.
aSMA	Abcam	ab5694
Albumin	Abcam	Ab19194
Epcam	Abcam	Ab32392
Krt19	Abbomax	A3190
Fah	HepatoScience	HS602-910
CD63	R&D	mab5417
CD56	proteintech	14255-1-AP
Hnf4a	Santa Cruz	sc-8987
F-actin	Molecular Probes	A12381
Pan-CK	Santa Cruz	sc-8018
Sox9	Millipore	ab5355
Lgr5	Abcam	ab75732
GFP	Molecular Probes	A-11120
Itgb4	Abcam	Ab29042
E-cadherin	Abcam	ab5303
Ki67	BD	550609
Ki67-FITC	eBioscience	11-5698
CD63-PE	Biolegend	143904
CD56-APC	R&D	FAB7820A

CD11-FITC	eBioscience	11-0112
CD45-FITC	BD	553079
CD68-FITC	Biolegend	137006

Table S2 List of reagents

Reagents	Source	Cat. No.
Tamoxifen	Sigma-Aldrich	T5648
3,5-diethoxycarbonyl-1,4-dihydrocollidin (DDC)	Sigma-Aldrich	137030
carbon tetrachloride (CCl4)	Innochem	A33986
corn oil	Innochem	A14155
2-(2-nitro-4-trifluoromethylbenzoyl)-1,3-cyclo-hexanedione (NTBC)	N/A	N/A
Collagenase D	Roche	11088858001
Hoechst 33342	Invitrogen	R37165
OptiPrep	Sigma-Aldrich	D1556

Table S3 List of primers

Genes	Forward Primer	Reverse Primer
Aat	TCGGAGGCTGACATCCACAA	TCAACTGCAGCTCACTGTCTGG
Alb	GGTGTGTTTCGCCGAGAAGCAC	GGCGGCAGACTCATCGGC
Krt19	GGGGGTTCAGTACGCATTGG	GAGGACGAGGTCACGAAGC
EpCAM	GCGGCTCAGAGAGACTGTG	CCAAGCATTTAGACGCCAGTTT
Gapdh	TGTGTCCGTCGTGGATCTGA	CCTGCTTCACCACCTTCTTGA
Hnf1b	GTCGCTCCAGCAAGAACTCC	GACGGCAGTAACTCCTCCAAG
Lgr5	GGAAATGCTTTGACACACATTC	GGAAGTCATCAAGGTTATTATAA
Prom1	CCTTGTGGTTCTTACGTTTGTTG	CGTTGACGACATTCTCAAGCTG
Sox9	AGTACCCGCATCTGCACAAC	ACGAAGGGTCTCTTCTCGCT
Itga3	CCTCTTCGGCTACTCGGTC	CCGGTTGGTATAGTCATCACCC
Dmbt1	TCAGCACAAGTCCTCCATCAT	TCCACAGGTGAGACTCATACC

Fabp1	ATGAACTTCTCCGGCAAGTACC	CTGACACCCCCTTGATGTCC
G6P	GAATTACCAAGACTCCAGG	TGAGACAATACTTCCGGAGG
Cps1	ACATGGTGACCAAGATTCCTCG	TTCCTCAAAGGTGCGACCAAT
Cyp7a1	GGGATTGCTGTGGTAGTGAGC	GGTATGGAATCAACCCGTTGTC
Gja1	TCCTTCAGATCATATTCGTGTC	GCAGCCATTGAAGTAAGCAT
Hnf4a	ACACGTCCCCATCTGAAGGTG	CTTCCTTCTTCATGCCAGCCC
Abcg2	TGAAACCTGGCCTTAATGCT	GTTCCGACCTTAGAATCTGCT
